# Modelling geospatial distributions of the triatomine vectors of *Trypanosoma cruzi* in Latin America

**DOI:** 10.1371/journal.pntd.0008411

**Published:** 2020-08-10

**Authors:** Andreas Bender, Andre Python, Steve W. Lindsay, Nick Golding, Catherine L. Moyes

**Affiliations:** 1 Big Data Institute, Li Ka Shing Centre for Health Information and Discovery, University of Oxford, Old Road Campus, Oxford, United Kingdom; 2 Department of Biosciences, Durham University, DH1 3LE, Durham, United Kingdom; 3 Department of BioSciences, University of Melbourne, Parkville, Melbourne, Victoria, Australia; Universidade do Estado do Rio de Janeiro, BRAZIL

## Abstract

Approximately 150 triatomine species are suspected to be infected with the Chagas parasite, *Trypanosoma cruzi*, but they differ in the risk they pose to human populations. The largest risk comes from species that have a domestic life cycle and these species have been targeted by indoor residual spraying campaigns, which have been successful in many locations. It is now important to consider residual transmission that may be linked to persistent populations of dominant vectors, or to secondary or minor vectors. The aim of this project was to define the geographical distributions of the community of triatomine species across the Chagas endemic region. Presence-only data with over 12, 000 observations of triatomine vectors were extracted from a public database and target-group background data were generated to account for sampling bias in the presence data. Geostatistical regression was then applied to estimate species distributions and fine-scale distribution maps were generated for thirty triatomine vector species including those found within one or two countries and species that are more widely distributed from northern Argentina to Guatemala, Bolivia to southern Mexico, and Mexico to the southern United States of America. The results for *Rhodnius pictipes*, *Panstrongylus geniculatus*, *Triatoma dimidiata*, *Triatoma gerstaeckeri*, and *Triatoma infestans* are presented in detail, including model predictions and uncertainty in these predictions, and the model validation results for each of the 30 species are presented in full. The predictive maps for all species are made publicly available so that they can be used to assess the communities of vectors present within different regions of the endemic zone. The maps are presented alongside key indicators for the capacity of each species to transmit *T. cruzi* to humans. These indicators include infection prevalence, evidence for human blood meals, and colonisation or invasion of homes. A summary of the published evidence for these indicators shows that the majority of the 30 species mapped by this study have the potential to transmit *T. cruzi* to humans.

## Author summary

The Pan American Health Organisation’s Strategy and Plan of Action for Chagas Disease Prevention, Control and Care highlights the importance of eliminating those triatomine vector species that colonise homes, and has had great success in many locations. Since indoor residual spraying campaigns have targeted these species, their importance relative to other vectors has diminished and their geographical distributions may also have changed. It is now vital to consider the full community of vector species, including previously dominant vectors as well as secondary or minor vector species, in order to target residual transmission to humans. Our aim was to define the geographical distributions of the most commonly reported triatomine species in the Chagas endemic region from northern Argentina and Chile to the southern United States of America. We extracted reports of triatomine vector species observed at specific locations from a public database and we used a geostatistical model to generate fine-scale predictive maps for thirty triatomine vector species. Data quality and data availability issues necessitate careful interpretation of the results, so (un)certainty intervals are presented alongside each map. We also present these maps alongside a summary of the published evidence for key indicators related to the capacity of each species to transmit the Chagas parasite to humans. This evidence shows that most of the 30 species that we have mapped pose a potential threat to human populations.

## Introduction

American trypanosomiasis, or Chagas disease, is one of the 10 neglected diseases addressed by the London Declaration, which calls for control and elimination of these devastating diseases by 2020 [1]. It is a disease where vectorial transmission occurs from northern Argentina and Chile to the southern United States of America, and a ‘Strategy and Plan of Action for Chagas Disease Prevention, Control and Care’ has been set out by the Pan American Health Organisation (PAHO) [2]. This strategy includes the elimination of domestic vectors to prevent intra-domiciliary transmission, as well as screening blood donors and pregnant women to prevent transmission via blood donation or the placenta, and implementation of best practice in food handling to prevent oral transmission. Our study focuses on the primary route of infection; the contamination of a vector bite with faeces of that vector.

The *Trypanosoma cruzi* parasite is transmitted to humans by over 150 different vector species from 18 different genera [3]. The transmission risk that each vector species poses is influenced by how likely it is that the species in question will come into contact with humans. The likelihood of human contact is influenced by short-distance movement (for example, whether the species enters and/or colonises homes) and the larger-scale geographical distribution of that species. Studies assessing vulnerability of individuals to Chagas disease have shown that, while housing, ecotype and socio-economics are all relevant, triatomine presence is the most important indicator [4]. Thus understanding the distribution of these vector species is vital to both target control measures and to assess disease risk.

Before the current intervention era, five vector species were recognised as being dominant in the transmission of *T. cruzi* to humans based on their habit of colonising houses, behaviour (feeding-defecation interval) and widespread geographical distributions [5]. Since indoor residual spraying (IRS) campaigns have successfully targeted these dominant species in many locations, their importance relative to other vectors has diminished and their geographical distributions may also have changed [6]. It is now vital to understand the full community of vector species, including previously dominant vectors as well as secondary or minor vector species, in order to target residual transmission to humans [6–8].

Several studies have investigated species behaviours that influence short distance travel in and around homes, such as host-seeking, aggregation and dispersal [9–19], but fewer studies have considered the larger-scale geographical distributions of these species. The studies of geographical species distributions that have been conducted typically focus on a single country or a region within a country [19–24]. One earlier study considered the distribution of triatomines infected with a virus across South America, without distinguishing species [25], and previous studies have mapped individual species across their ranges [26–31], but no studies have considered the geographical distributions of multiple, individual dominant and secondary vector species across the Chagas endemic region from northern Argentina and Chile to the southern United States of America. A lack of consistent region-wide information makes it harder to construct an overview for the region as a whole or to compare areas within the endemic zone.

The data recording presence of a species are often sparse and suffer from sampling bias, which makes inter-region comparison of these records difficult. The aim of this study is to use statistical models to produce a comprehensive set of maps predicting the distributions of triatomine vector species while taking into account the limitations of the data. We use an extensive database of reported occurrences of each species, and data on environmental variables that are likely to influence species presence, and we build species distribution models to improve our current understanding of the spatial distribution of vectorial transmission of *T. cruzi*.

## Materials and methods

### Study area

The study area was defined as the Chagas endemic region, which extends from northern Argentina and Chile to the southern United States of America. The study area for each individual species was defined as the area encompassing all reports of that species since the year 2000 plus a buffer zone of 5 degrees (approximately 300km).

### Species occurrence and background points

The primary source of vector species data was a database of vector occurrence locations, which was supplemented with additional species presence points derived from a database of infections in vector species. Data on vector occurrence was extracted from DataTri, a publicly available database that reports the presence of a given triatomine species, the date of collection (if available) and geographical coordinates for each collection [32]. Additional vector occurrence data was added using a database of *T. cruzi* infections in triatomines that also provided the vector species found, the date of collection (if available) and geographical coordinates for each collection [33]. Any data points from DataTri that were duplicated in the second data set were removed before vector occurrence data from the infections database was added to the DataTri data set. Data points before the year 2000 were removed because the aim was to investigate vector distributions in the current era.

The available vector occurrence data is usually referred to as *presence only* data. Techniques for modelling such data often involve augmenting the presence data with *pseudo-absence* or *background* points, which requires a source of appropriate background data [34–36].

Here we use a target-group background (TGB) approach by choosing background data that exhibits similar sampling bias as the occurrence data [37]. This approach can reduce the bias introduced by preferential sampling of the presence locations. It was successfully used to map geographical distributions of malaria hosts and vectors [38] and predict infection risk zones of yellow fever [39]. In simulation studies this method also performed well when compared to approaches using presence-absence data [37, 40]. As with all models of presence-only data, the maps produced using the TGB approach represent relative rather than absolute probabilities of species occurrence.

**Fig 1:**
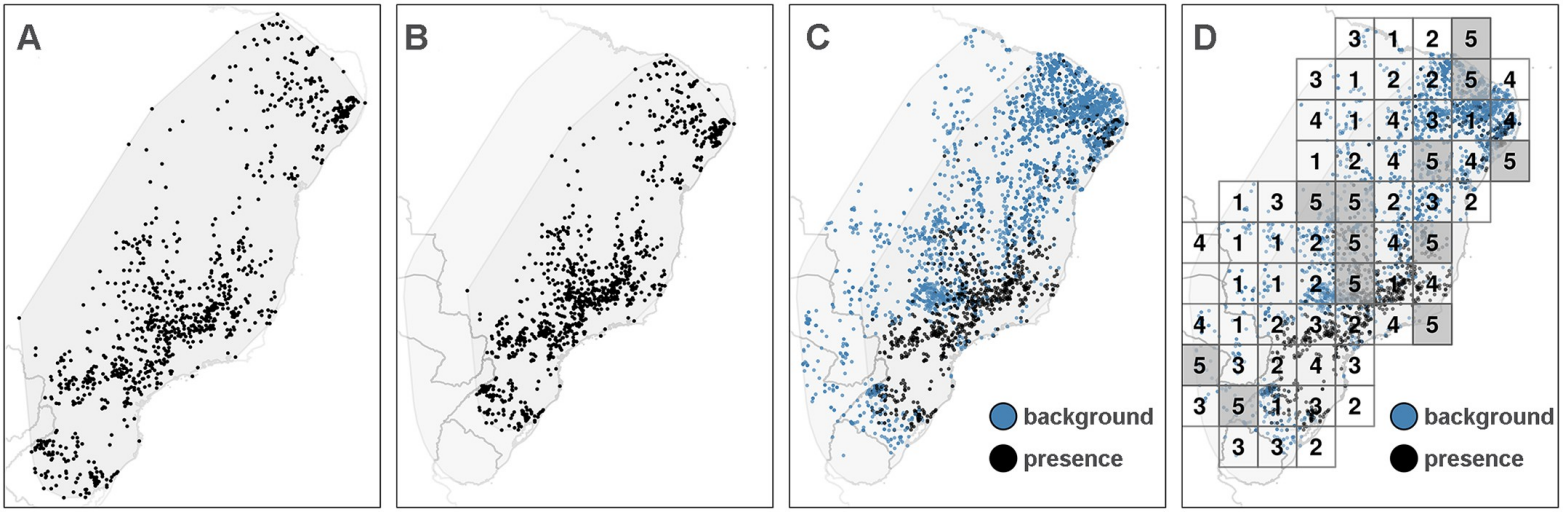
Construction of background points. Illustration of the construction of background points using the TGB approach for species *Panstrongylus megistus*. Panel (A): A convex hull is constructed around the presence locations of the species. Panel (B): The hull is extended by a fixed width of 5 degrees (*extended hull*). Panel (C): Background points are added using presence locations of all other species within the extended hull. Panel (D): Blocks of width *w*_*k*_ are allocated randomly across the extended hull. The observations in blocks numbered 1-4 are used as training data, and the observations in blocks numbered 5 are assigned to the test data. One fold consists of all blocks sharing the same number. Figure created by the authors using R package **tmap** [41].

We constructed one TGB dataset for each vector species as outlined below and illustrated in Fig 1 for *Panstrongylus megistus*:

1. The presence locations of vector species *k* = 1, …, *K* (target-group) were extracted from the database and a convex hull containing all presence locations was constructed (panel (A) in Fig 1).
2. This hull was extended by a constant width of 5 degrees in all directions to allow for uncertainty with respect to the range of the species being modelled (*extended hull*; panel (B) in Fig 1).
3. The presence locations of all other species within the extended hull were defined as background points (blue dots in panel (C) of Fig 1).
4. Duplicate observations at the same site and the same year were removed.
5. At the modelling stage, the background points were weighted such that their total weight is equal to the number of presence observations (cf. [37, 38]).

The weighting of background points means that if presence and background points were randomly distributed the predicted relative probability would be about 0.5. Thus probabilities > 0.5 indicate that it is likelier to observe presence than background, rather than an absolute probability of occurrence.

For some species, only few observations were available in the dataset. It was suggested that approximately five [42] or ten [43–45] events (presences) per predictor are required to reliably fit a logistic regression. Given that we use up to 30 predictors, this would imply a sample size of *n* ≈ 150 and ≈ 300, respectively. In our data, 14 and and 9 species fulfilled this (approximate) requirement. For completeness, we fit models for all species with *n* > 50, but obviously results must be interpreted with care as the sample size (number of presence observations) decreases (see Results section). Species with fewer than fifty observations in the training and test data were not modelled, however, their presence locations were used as background points for the species that were modelled.

### Environmental variables

Previous work has shown that vector distributions are influenced by climate, land cover types and rural/urban classifications [19–24]. Environmental variables for these three data types were obtained at a resolution of 5 × 5 kilometres. The climatic variables used were *land surface temperature* (annual; day, night and diurnal difference) [46], two measures of *surface moisture* (annual) [47], *rainfall* (annual) [48], *elevation* (static) and *slope* (static) [49]. The variables used for land cover were the 16 IGBP *land cover* classes (annual) [50] and an *enhanced vegetation index* [51]. Finally the variables used to distinguish rural, peri-urban and urban areas were *urban footprint* (static) [52], *nighttimelights* (static) [53], *human population* (annual) [54] and *accessibility* (static, based on road networks and distance to cities) [55]). Annual environmental variables were not always available for all time periods in which occurrence data was available. In this case, we instead used values from the closest year. A full description of each variable is given in the supplement (Table A, S1 File).

### Model evaluation

In the context of spatial analysis, data available for modelling often only encompasses few locations in areas for which predictions are generated. Therefore, the model is usually evaluated on out of sample data to avoid over-fitting, to ensure transferability to new locations and to obtain realistic estimates for the goodness of fit. Standard approaches to model evaluation, however, can yield over-optimistic metrics of the model predictive ability unless the spatial nature of the data (and the model) is taken into account [56–58]. To address these concerns, the data was initially split randomly into *train-test data* (80%) and *evaluation data* (20%), stratified by species. The latter dataset is not utilised during model building but later used to evaluate the models ability to interpolate and the final prediction. Additionally, the train-test data was split into five folds. Following recommendations in [59] each fold consisted of multiple spatial blocks, where the block size *w*_*k*_ for species *k* = 1, …, *K* was set such that approximately 50 blocks (10 per fold) would cover the extended hull of that species and defined as $w_{k}=\sqrt{\frac{a_{k}}{50}}$, where *a*_*k*_ is the area of the extended hull of species *k*. Fig 1 (panel (D)) depicts the resulting blocks and folds for species *Panstrongylus megistus*. For each species, blocks one through four were assigned to the training data, while blocks numbered five (grey shade) were only used to obtain out-of-sample test errors. Allocation of blocks was spatially random to avoid systematic bias of presence and background locations in any of the folds, but stratified with respect to species presence such that the proportion of presence and background points was approximately equal in all folds. The spatial blocking for all species considered in our analyses are provided in [60]. Model performance was evaluated by the area under the receiver operator curve (AUC), which measures the models ability to discriminate between presence and background points. After model evaluation as reported in the Results section was complete, the best model was refit on all data for the final prediction.

### Modelling

To estimate the triatomine species distributions we fit a logistic regression to the target-group background (TGB) data using a generalised additive model (GAM). This modelling framework is comprised of two components: observations (1) and a linear predictor (2). We consider a Bernoulli process to model the background/presence (*y*_*k*,*t*,*i*_ ∈ {0, 1}) of each species *k* in year *t* ∈ {2000, …, 2016} at location $s_{i}\in E_{k}$. Within this framework, we specify the Bernoulli model

$$\begin{array}{c} y_{k,t,i}∼Bernoulli\left(\pi_{k,t,i}\right),\;\;\;\;i=1,\ldots ,n_{k}, \end{array}$$ (1)

where *i* = 1, …, *n*_*k*_ are the observations per species. For each species *k* = 1, …, *K*, we set a spatial domain delimited by the extended hull $E_{k}\in R^{2}$ formed by the spatial locations of the corresponding species (see Fig 1 for further detail). The relative probability of occurrence *π*_*k*,*t*,*i*_ is estimated by a logistic GAM with linear predictor (2)

$$\begin{array}{c} \eta_{k,t,i}=\text{log}\;(\frac{\pi_{k,t,i}}{1-\pi_{k,t,i}})=\beta_{0}+\sum_{p=1}^{P_{k}}f_{k,p}\left(x_{p,t,i}\right)+GP_{k,i}\left(\ell \right),\;\;\;\;i=1,\ldots ,n_{k}, \end{array}$$ (2)

where *f*_*k*,*p*_(*x*_*p*,*t*,*i*_) is the species specific, potentially non-linear, effect of the *p*-th covariate estimated by a penalised thin-plate spline [61] and *GP*_*k*,*i*_(*ℓ*) is a two-dimensional, species-specific Gaussian process (GP) with range parameter *ℓ* evaluated at location *s*_*i*_ (the smoothness parameter was set to 1.5). The number of covariates *P*_*k*_ can vary by species as some of them might not have enough unique values within the spatial extent of the species to be relevant for analysis. Here, covariates were only included if the number of unique values was at least twenty. The correlation function of the GP was defined by *C*(*x*, *x*′) = *ρ*(||*x* − *x*′||), where *ρ*(*d*) = (1 + *d*/*ℓ*) exp(−*d*/*ℓ*) is the simplified Matérn correlation function with range parameter *ℓ* = *max*_*ij*_||*x*_*i*_ − *x*_*j*_|| as suggested in [62] and implemented in [63].

The model was estimated by optimising the penalised restricted maximum likelihood (REML) criterion (3)

$$\begin{array}{c} D\left(\psi \right)+\gamma (\phi \left(\psi \right)+\phi^{*}\left(\psi \right)) \end{array}$$ (3)

using a double shrinkage approach where *ψ* is a vector of all coefficients associated with the smooth functions *f* and *GP*, *D*(*ψ*) is the model deviance and *ϕ*(⋅) and ϕ*(⋅) are range space and null space penalties of the model coefficients *ψ* [61, 64]. The first penalty (range space) controls the smoothness of functions *f*_*k*,*p*_ and *GP*_*k*_, while the second penalty (null space) enables the removal of individual terms from the model entirely. The *γ* parameter can be used to globally increase the penalty and thus to obtain smoother, sparser and therefore potentially more robust models. Practical estimation was performed using techniques introduced in [65–67] to increase computational speed and reduce memory requirements.

Six model specifications (Table 1) were considered for this analysis, varying by the definition of covariate effects in Eq 2 and whether the global GP term *GP*_*k*,*i*_(*ℓ*) was included. For each species, the final model (out of the six candidate models in Table 1) was selected based on its performance (AUC) on the test data (fold 5). Model 1 has no tuning parameters and was fit directly to the complete training data (folds 1-4). Models 2 through 6 were first tuned with respect to the global penalty *γ* ∈ {1, …, 4} based on 4-fold cross-validation on folds 1 through 4. Based on the value of *γ* that yielded the highest average AUC, the models were refit on the complete training data (blocks 1–4). These models were used to calculate the out-of-sample extrapolation and interpolation error (see “Results” section for details). After model evaluation the best model for each species was refit on all data (blocks 1 through 5 and the random hold-out data) to create final predictions.

**Table 1:** Model specifications considered in the analysis.

Model specification	Covariate effect definition	GP (global)
1	linear effects *f*_*k*,*p*_(*x*_*p*_) ≔ *β*_*k*,*p*_ ⋅ *x*_*p*_	No
2		Yes
3	$f_{k,p}\left(x_{p}\right)≔\sum_{m=1}^{10}B_{p,m}\left(x_{p}\right)\psi_{k,p,m}$, where *B*_*p*,*m*_(*x*_*p*_) and *ψ*_*k*,*p*,*m*_ are spline basis functions and coefficients	No
4		Yes
5	spatial (bivariate) varying coefficient model *f*_*k*,*p*_(*x*_*p*_) ≔ *GP*_*k*,*p*_(*ℓ*) ⋅ *x*_*p*_	No
6		Yes

### Implementation

All calculations were performed using the **R** language environment [68]. Thematic maps were created using package **tmap** [41]. Data munging and pre-processing was performed using packages **dplyr** [69] and **tidyr** [70]. Spatial cross-validation was set up using package **blockCV** [71]. Package **mgcv** was used to fit the GAMs [63].

### Vectorial capacity of the mapped species

For each triatomine species that was mapped, information related to its importance in transmitting *T. cruzi* to humans was collated. The prevalence of infection with the *T. cruzi* parasite was calculated using the data from an existing repository [33]. Collections of less than twenty individuals of a species were excluded and the mean prevalence was calculated for all species where the remaining number of collections exceeded ten. Relevant behavioural data for each vector species was extracted from the published literature.

## Results

### Species distributions

A total of 30 species were mapped. *Triatoma infestans* is predicted to occur from northern Argentina and Chile to southern Bolivia and Peru, overlapping in part with the predictions for *Triatoma guaysayana*, although *T. guaysayana* isn’t predicted in Peru. *Panstrongylatus lutzi*, *Psammolestes tertius*, *Rhodnius nasutus*, *Rhodnius neglectus*, *Triatoma brasiliensis* and *Triatoma psuedomaculata* are all predicted to primarily occur within Brazil. *Triatoma sordida* is predicted to occur in Brazil, Paraguay and Bolivia while *Triatoma rubrovaria* is mainly predicted to occur in Uruguay. *Eratyrus mucronatus*, *Panstrongylatus geniculatus*, *Panstrongylatus rufotuberculatus*, *Rhodnius pictipes* and *Rhodnius robustus* are predicted to overlap to differing degrees across a broad area that encompasses northern Bolivia and Peru, northwestern Brazil, Ecuador, Colombia, Venezuela, Guyana, Suriname and French Guiana. *Triatoma maculata* predictions are restricted to the northern part of this area, while *Rhodnius prolixus* is predicted even further north in Colombia and Venezuela, and *Panstrongylatus chinai* is only predicted at the far west of this area within Peru and Ecuador. The predicted distributions of *Panstrongylatus geniculatus*, *Panstrongylatus rufotuberculatus*, *Rhodnius pallescens* and *Triatoma dimidiate* all extend from northern South America to Central America. *Triatoma barberi*, *Triatoma longipennis*, *Triatoma mazzotti*, *Triatoma Mexicana* and *Triatoma pallidipennis* were all predicted to occur in Mexico only. *Triatoma gerstaeckeri*, *Triatoma protracta* and *Triatoma rubida* were predicted to occur from northern Mexico to the southern United States of America and *Triatoma sanguisuga* was predicted to occur exclusively in the southern United States of America.

A summary for all species that were modelled is provided in Table 2, including the specification of the model selected on training data as well as the AUC of this model evaluated on test data (fold 5) and the AUC obtained on the 20% randomly selected hold-out data (denoted by AUC*). The former is an indicator of the model’s transferability and ability to predict into new areas with potentially unseen covariate values or combinations, within the area that was modelled (cf. Fig 1). This is important because this is precisely the goal of the TGB approach and other modelling strategies that account for preferential sampling. The latter value indicates how well the model interpolates.

**Table 2:** Summary table for all species considered in the analysis ordered by number of presence observations.

species	n presence (test)	n background (test)	Selected model (*γ*)	AUC	AUC*
***Triatoma infestans***	2499 (430)	4458 (896)	5 (3)	0.96	0.97
Triatoma dimidiata*	1186 (154)	2883 (787)	6 (4)	0.97	0.93
*Panstrongylus megistus*	968 (213)	3409 (827)	6 (1)	0.83	0.89
*Triatoma brasiliensis*	820 (151)	1989 (346)	2 (1)	0.69	0.81
Triatoma sordida*	809 (139)	4919 (768)	5 (2)	0.83	0.89
*Triatoma pseudomaculata*	804 (239)	3100 (687)	4 (1)	0.73	0.81
*Triatoma barberi*	455 (56)	2161 (612)	4 (1)	0.88	0.92
*Triatoma mexicana*	430 (44)	2046 (456)	6 (2)	0.92	0.96
*Rhodnius prolixus*	319 (42)	962 (229)	4 (2)	0.64	0.85
***Panstrongylus geniculatus***	282 (39)	6425 (824)	3 (1)	0.87	0.82
Triatoma longipennis*	282 (25)	1974 (324)	2 (2)	0.97	0.95
***Triatoma gerstaeckeri***	280 (91)	2480 (482)	2 (1)	0.85	0.96
*Triatoma protracta*	269 (42)	2092 (212)	6 (1)	0.89	0.97
Triatoma pallidipennis*	256 (15)	2286 (265)	2 (2)	0.96	0.96
*Panstrongylus lutzi*	136 (8)	2965 (609)	1 (–)	0.78	0.92
***Rhodnius pictipes***	130 (25)	3542 (940)	5 (1)	0.91	0.91
*Triatoma rubida*	126 (23)	1913 (388)	4 (1)	0.98	0.95
*Rhodnius neglectus*	122 (18)	3993 (655)	4 (1)	0.93	0.95
Triatoma mazzottii*	105 (7)	2531 (581)	4 (1)	0.8	–
*Rhodnius pallescens*	93 (21)	898 (202)	2 (1)	0.78	0.89
Triatoma guasayana*	92 (8)	2761 (493)	1 (–)	0.52	0.75
*Triatoma rubrovaria*	92 (11)	572 (227)	3 (1)	1.00	0.95
*Rhodnius robustus*	90 (22)	3395 (915)	4 (1)	0.97	0.94
*Triatoma sanguisuga*	89 (17)	701 (173)	5 (2)	1.00	0.92
*Psammolestes tertius*	75 (17)	3969 (825)	2 (3)	0.79	0.90
Eratyrus mucronatus*	67 (13)	2495 (656)	5 (4)	0.80	0.84
*Triatoma maculata*	65 (10)	907 (169)	5 (3)	0.8	0.80
*Panstrongylus chinai*	57 (7)	209 (53)	4 (1)	0.8	–
*Rhodnius nasutus*	56 (10)	2178 (423)	2 (1)	0.90	0.83
Panstrongylus rufotuberculatus*	55 (13)	3262 (608)	3 (3)	0.66	0.61

**Fig 2:**
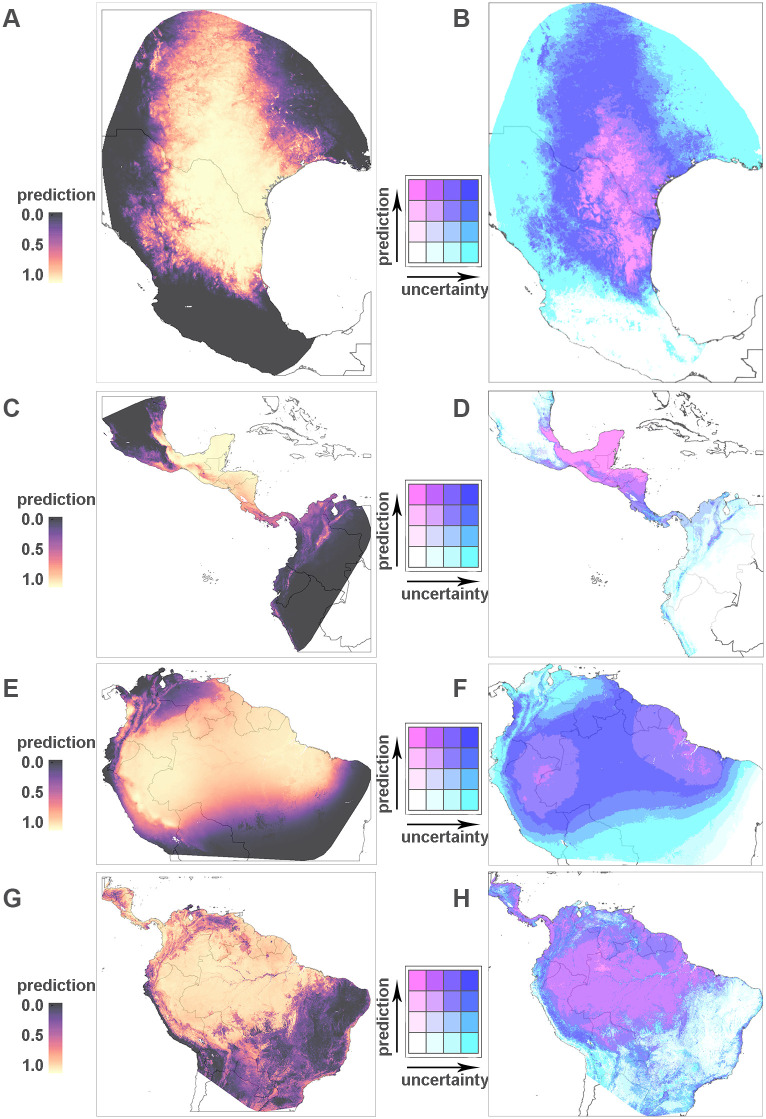
Predicted relative probability of occurrence. Predictions for 4 selected species at a resolution of 5 × 5 km within the respective extended hull of species occurrence (left panel) and bivariate map (right panel), where darker colours indicate higher predicted probabilities while the transition from white/pink to turquoise/blue indicates increased uncertainty. Row 1 (A, B): *Triatoma gerstaeckeri*; row 2 (C, D): *Triatoma dimidiata*; row 3 (E, F): *Rhodnius pictipes*; row 4 (G, H): *Panstrongylus geniculatus*. Figure created by the authors using R package **tmap** [41].

**Fig 3:**
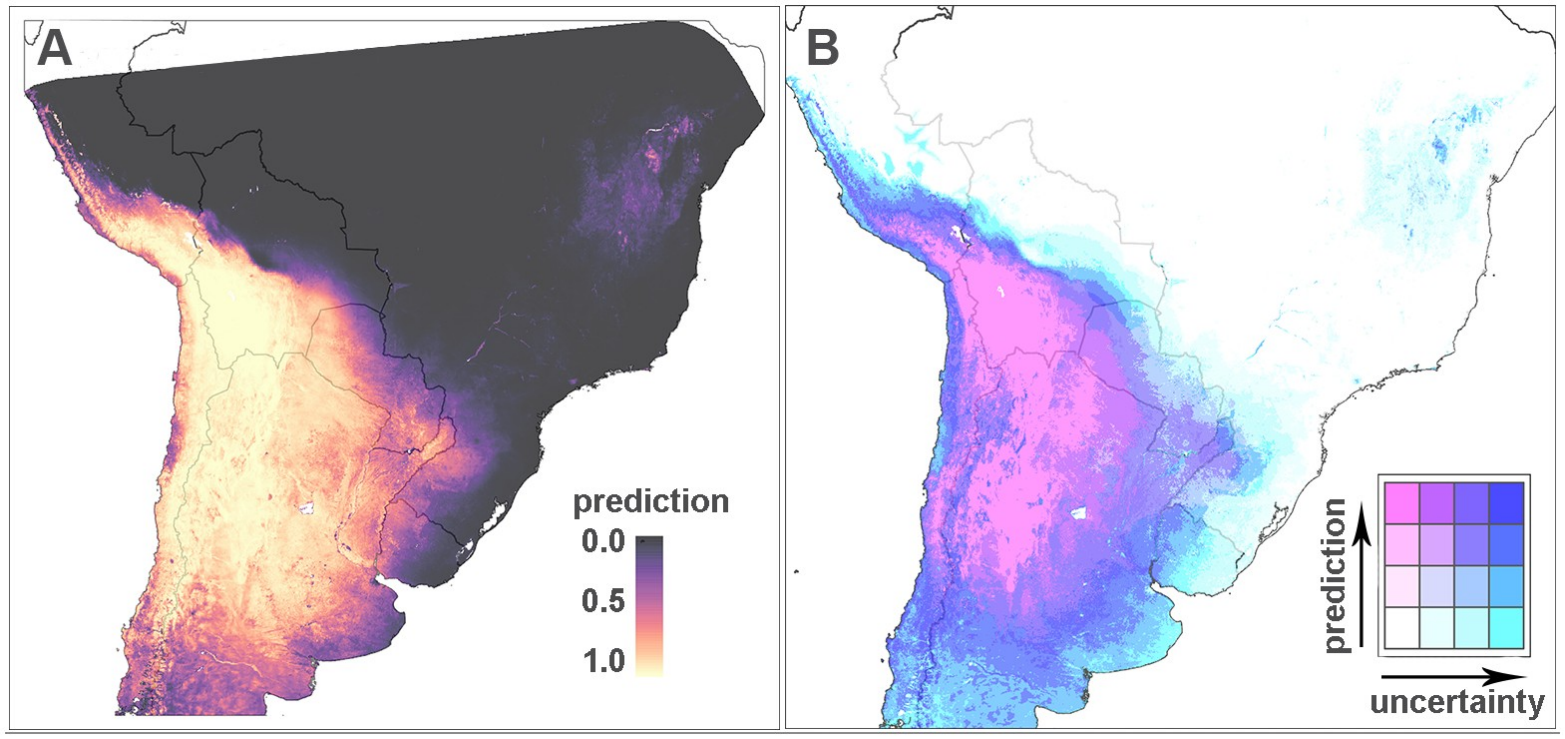
Left panel: Final predicted map for *T.infestans*. Right panel: A bivariate map of the predictions that indicates areas of high vs. low probabilities together with the model uncertainty. Darker colours indicate higher predicted probability. Transitions from white/pink to turquoise/blue indicate higher uncertainty.

Summary table for thirty species for which predictive maps were created, ordered by number of occurrence observations. Species highlighted in bold are shown in Figs 2 and 3, rasters of all 30 species (including 95% CI) and map images are given in [72] and [73], respectively. Asterisks (*) mark species for which the block width *w*_*k*_ used to construct spatial blocking was smaller than a preliminary estimation of the range of spatial auto-correlation, thus estimated AUC values might be overoptimistic in these cases. Column “Selected model” indicates the model specification that was selected based on its performance on the training data and refers to the model specifications defined in Table 1. If applicable, *γ* indicates the value of the selected global penalty multiplier (cf. Eq (3)). The reported AUC value was calculated on the test data set (fold 5 in Fig 1). The AUC* value was calculated on the 20% hold-out data (not spatially blocked). Entries “–” indicate that there were not enough observations and/or unique predicted values in the hold-out data for calculation.

The AUC values were all well above the 0.5 random classification threshold (mean: 0.85, SD: 0.12), indicating the maps usefulness to identify areas of higher probability of presence relative to background points. The comparatively low AUC values for species *T. brasiliensis* and *T. pseudomaculata* could be partially due to an overlap with many other species, thus making it difficult to discriminate between presence and background. The AUC* values were on average higher and had a lower variance (mean: 0.88, SD: 0.08) but generally consistent with the AUC values obtained on the spatial hold-out test data (fold 5). The predicted values (including 95% CI) in raster format for all species listed in Table 2 are given in [72], (.gri file format). Respective visualisations, i.e. map images, are available from [73].

#### Predicted distributions of four example species

In this section, we present the results for four diverse species that provide examples from different genera including dominant and secondary vectors with domestic, peri-domestic and sylvatic habits. In addition, we provide detailed discussion of the results for one of the most important of these vectors, *T. infestans*, in the following section. Together distributions of these species cover most of the endemic region from northern Argentina and Chile to the south of the United States. Predictions are presented alongside bivariate maps that display prediction and (un)certainty in one map. To do so, predictions and uncertainty (defined by the width of confidence intervals) are divided into intervals (here [0, .25), [.25, .5), [.5, .75), [.75, 1], and [0, .075), [.075, .15), [.15, .3), [3, 1], respectively. The cut off for uncertainty was chosen because a CI width of ≥ .3 means that the upper and lower CIs fall into different categories of the probability intervals. The legend in the bivariate maps indicates which colours correspond to which combination of prediction and uncertainty.

*Triatoma gerstaeckeri* colonises homes and kennels and is known to bite humans (S2 File). The predicted distribution of this species from the southern USA through much of Mexico is shown in Fig 2a. Uncertainty in these predictions is high at the northern boundaries of this species where sampling is particularly sparse (Fig 2b and [60]). *Triatoma dimidiata* colonises homes, as well as sylvatic and peri-domestic habitats (S2 File), and is a dominant vector of *T. cruzi*. It’s distribution predicted using data for the time period from 2000 onwards ranges from southern Mexico through Central America into northern Venezuela and Colombia (Fig 2c and 2d). Predictions are high with low uncertainty in Central America whereas predictions are lower with higher uncertainty in northern Venezuela and Colombia. It is important to note that the block width for this species is smaller than an estimation of the range of spatial auto-correlation, thus the AUC values may be optimistic. *Rhodnius pictipes* colonises palm trees and has been implicated in the contamination of products for human consumption (S2 File). The predicted distribution of *R. pictipes* in northern Brazil and Bolivia, French Guiana, Suriname, Guyana, Venezuela, Colombia, Ecuador and Peru is shown in Fig 2e. It is important to note the areas of high uncertainty within the region of higher predicted probability of presence for this sylvatic species (Fig 2f). The confidence intervals for this species are higher than those seen for *Panstrongylus geniculatus* in the same region, which reflects the lower volume of data available for *R. pictipes*. *Panstrongylus geniculatus* colonises trees and rodent nests, has been found in urban areas, and is known to bite humans (S2 File). Its predicted distribution, which overlaps with that of *R. pictipes* in South America and extends further north as far as Honduras in Central America, is shown in Fig 2g. The uncertainty in these predictions is typically low but is higher at the fringes of the area where the predicted relative probability of presence is high (Fig 2h).

#### Distribution of *Triatoma infestans*

Arguably, the most important *T. cruzi* vector species is *T. infestans* making it a key target for indoor residual spraying campaigns that have the potential to alter the distribution of this predominantly domestic species. Intervention coverage data was not available to our models so it is particularly important to consider the uncertainty in the predictions for *T. infestans*. Fig 3 shows the predicted probabilities for this vector species using data from the year 2000 onwards (left panel) alongside a bivariate map that highlights the (un)certainty of the estimation (right panel). The model predicts areas of high relative probabilities of presence with higher certainty in southern Bolivia, northern Chile and northwestern Argentina, which aligns well with the observed presence points. The uncertainty is usually high in unsampled areas, e.g., along the border of Argentina and Chile, and eastern parts of Paraguay. Low probabilities of occurrence are predicted in Brazil and northeastern Paraguay, with varying levels of certainty.

### Ranking importance of the environmental variables

Caution is needed when drawing conclusions from information on which environmental variables were selected by each model because i) many of these variables are highly correlated, for example temperature, rainfall, surface wetness and elevation, and ii) some important variables may not have been made available to the models as discussed above. It is, however, interesting to note the three variables selected as most important by each species model and these rankings are given in Table D, S3 File. Forest cover was one of the three most important variables for three species and *Eratyrus mucronatus*, *Panstrongylus rufotuberculatus* and *Rhodnius pictipes* are all known to colonise trees (S2 File). Eight other species models selected other vegetation cover variables as important and variables that define the urban-rural gradient (urbanicity, accessibility and human population) were selected as being among the most important by seven species models. Looking across all species models (Table E and Figure A, S3 File), the most important variables for predicting *T. cruzi* vector species distributions were temperature, the Gaussian process (the spatial component), evergreen broadleaf forest cover, the vegetation index, rainfall and elevation, followed by variables defining the urban-rural gradient and other types of vegetation cover. Nine land cover classes were never selected by any model. Unsurprisingly these were water, needleleaf forest cover, built up areas (information that was provided to the model by other variables that were selected), snow and ice, barren areas and unclassified land (which is a rare occurrence in the land cover data). For most species there are rarely single covariates with a contribution of more than 50%, meaning that each prediction is comprised of smaller contributions from many variables (Figure A, S3 File).

### Vectorial capacity of the mapped species

The current state of knowledge on factors related to the capacity of each of the mapped species to transmit *T. cruzi* to humans (vectorial capacity) is summarised in Table 3. Specifically, mean infection prevalence, confirmation of human blood meals in natural vector populations, and confirmation of colonisation or invasion of homes (including in urban areas) are listed. Less information is available on the feeding-defecation interval or defecation location for each species, and these values may be influenced by the different experimental conditions used, so these variables are not included in Table 3 but sources of evidence are listed in the supplement (Table B, S2 File). The 30 most commonly reported species mapped here encompass the five most important dominant vectors that frequently colonise homes (*P. megistus*, *R. prolixus*, *T. brasiliensis*, *T. dimidiata* and *T. infestans*) as well as species that often colonise peridomestic habitats such as chicken coops, rats nests, boundary walls, wood piles, palm trees, and livestock housing. These species encompass a range of mean *T. cruzi* infection prevalences from 0.8% in *T. sordida* to 55.6% in *T. longipennis*, although for 13 of the most commonly reported species there was insufficient data to generate a reliable mean infection prevalence value. In addition to differences among the mean values for species, there is also considerable variation within each species likely resulting from heterogeneities in factors such as intervention deployment and local host species. When viewing the summaries in Table 3, it is important to note that not all regions or species have been sampled or tested equally and a lack of published evidence for a specific component of vectorial capacity cannot be taken as definitive evidence of its absence. For example, no infections have been reported in *Eratyrus mucronatus* or *Psammolestes tertius*, but only 28 and 143 individuals have been tested, respectively, compared to 335,467 *T. sordida* individuals. In addition to the five important dominant vectors, there is evidence that many of the species mapped in this work are potential vectors of *T. cruzi*. Almost all of the 28 species that have been found to be infected with *T. cruzi* are known to invade homes, and at least 17 have been found to have fed on humans (Table 3). The sources of evidence—130 published articles in total—are given in the supplement (Tables B and C, S2 File).

**Table 3:** Infection prevalence and behaviour of selected species.

Species	Mean percent infected (n)	Human blood meals	Colonises homes	Invades home	Urban areas
*Eratyrus mucronatus*	NIF	-	yes	yes	-
*Panstrongylus chinai*	yes	-	yes	yes	-
*Panstrongylus geniculatus*	7.6 (46)	yes	-	yes	yes
*Panstrongylus lutzi*	6.8 (27)	yes	-	yes	-
*Panstrongylus megistus*	8.3 (261)	yes	yes	yes	yes
*Panstrongylus rufotuberculatus*	yes	-	yes	yes	-
*Psammolestes tertius*	NIF	-	-	-	-
*Rhodnius nasutus*	8.5 (73)	-	-	yes	-
*Rhodnius neglectus*	4.0 (120)	yes	-	yes	yes
*Rhodnius pallescens*	yes	yes	-	yes	-
*Rhodnius pictipes*	24.6 (36)	yes	-	yes	yes
*Rhodnius prolixus*	9.0 (10)	yes	yes	yes	-
*Rhodnius robustus*	25.9 (19)	-	-	yes	-
*Triatoma barberi*	yes	-	yes	yes	yes
*Triatoma brasiliensis*	3.2 (918)	yes	yes	yes	-
*Triatoma dimidiata*	28.9 (24)	yes	yes	yes	-.
*Triatoma gerstaeckeri*	yes	yes	yes	yes	-
*Triatoma guasayana*	yes	yes	-	yes	-
*Triatoma infestans*	27.6 (61)	yes	yes	yes	-
*Triatoma longipennis*	55.6 (14)	-	yes	yes	yes
*Triatoma maculata*	18.4 (18)	yes	-	yes	yes
*Triatoma mazzottii*	yes	-	-	yes	-
*Triatoma mexicana*	yes	-	-	yes	-
*Triatoma pallidipennis*	4.8 (17)	yes	yes	yes	yes
*Triatoma protracta*	yes	-	yes	yes	yes
*Triatoma pseudomaculata*	2.7 (988)	yes	yes	yes	yes
*Triatoma rubida*	yes	-	yes	yes	yes
*Triatoma rubrovaria*	3.1 (17)	-	-	yes	yes
*Triatoma sanguisuga*	yes	yes	-	yes	yes
*Triatoma sordida*	0.8 (1407)	yes	yes	yes	yes

The mean infection prevalence is given together with the number of triatomine collections that contributed to the mean. If there were insufficient data to calculate the mean, i.e. fewer than 10 collections of ≥ 20 individuals, then records of infected individuals (yes) or no infections found (NIF) are noted. Instances where there was no evidence for a particular behaviour are denoted by “-”.

## Discussion

This study models the contemporary geospatial distributions of the thirty most commonly reported triatomine species and putative vectors of the *T. cruzi* parasite to humans. Our approach allows the distributions of these different species to be compared, and to be overlaid, which increases our understanding of the community of vector species at different locations in the current intervention era.

Our aim was to consider the most commonly reported species in the Chagas endemic zone. To provide policy makers, stakeholders and researchers with relevant information, we included all species for which distribution maps could be reasonably estimated. However, as can be seen from Table 2, the training (and test) data for many species contained fewer than 300 or even fewer than than 150 presence observations reported since the year 2000. AUC values will tend to be less robust and potentially over- or under optimistic as sample size decreases. In these instances it is particularly important to take into account the uncertainty of the estimates as presented here. There are also locally important species for which maps could not be produced because they are only found within areas where the relevant surveillance records are not publicly available or because their range is limited so only small numbers of observations exist. For example, *Rhodnius ecuadoriensis* is an important vector in Ecuador [74] but the databases used in this study only provided 11 and 23 records, respectively, for known collection dates after the year 2000.

Earlier studies [19, 20, 22, 23, 25, 27, 28, 30, 31], most notably [24, 26], have modelled the distributions of some of these species but often previous work has focused on specific regions, states or countries. Additionally, comparisons with the previous work are limited because of differences in methodology, datasets and spatial extent under consideration. Only visual comparison is possible in most cases because the predicted values generated by previous studies are not openly available, precluding quantitative assessment of the different versions. Known *T. dimidiata* presence locations are predicted by the model presented here, but with higher probabilities for the locations in central America compared to Colombia and Ecuador. This may imply differentiation between these populations, for example, the subspecies *T. dimidiata capitata* is only found in Colombia, however, the subspecies *T. dimidiata dimidiata* is common to central America and Ecuador [75]. Within Central America, our predictions align well with a *T. dimidiata* map published in 2010, predicting this species throughout Central America extending up both the east and west coasts of Mexico [31]. This earlier map also provides a main and maximum distribution for *T. infestans*. The main distribution from the 2010 map (from La Rioja and northern Cordoba in Argentina up to Santa Cruz and southern Beni in Bolivia, as well as an area around Moquega in Peru) falls within our area of highest predictions for occurrence with moderate certainty (Fig 3), but an area of higher certainty can be seen running directly west of the previously published main distribution in Argentina and Bolivia, joining the main distribution predicted in Peru in the 2010 work. Comparisons with a *T. infestans* map published in 2002 [30] demonstrate the dramatic changes in Brazil over the last decades. The area of lowest probability of occurrence with highest certainty in our current map aligns with the area of absence in the 2002 map, whereas the areas in southwestern Brazil with low probability of occurrence but higher uncertainty in our current map align with areas of species presence in the 2002 map from Paraiba down to Mato Grosso do Sul and Rio Grande do Sul. These areas, where this species is predicted to be no longer present by both the 2010 study [31] and our current work, match PAHO reports of the Southern Cone Initiative (or INCOSUR). Hernandez *et al*. [22] modelled the joint distribution of *T. infestans* and *Mepraia spinolai* in the Coquimbo, Valparaíso and Metropolitana Regións of Chile, which are all regions where our model predicted high relative probability of occurrence of this species. Ceccarelli *et al*. [25] generated climatic suitability maps for infected *T. infestans* triatomines using two climatic datasets, the Advanced Very High Resolution Radiometer onboard the National Oceanic and Atmospheric Administration meteorological satellite series (AVHRR) and the WorldClim dataset. Their AVHRR results are closest to our distribution maps but the studies cannot be compared directly due to the different outcomes modelled, i.e. vector occurrence and infected vector occurrence.

In general, our predicted maps show good agreement with respect to regions that highlight higher vs. lower probabilities of occurrence when compared to earlier studies. Curtis-Robles *et al*. [19] recently investigated the spatial distribution of, among others, *T. gerstaeckeri*,*T. sanguisuga* and *T. rubida* within the state of Texas in the USA and their areas of species occurrence match our areas of high relative probability of occurrence with high certainty for these three species within this region. Garza *et al*. [23] also mapped the distribution of *T. gerstaeckeri* and predicted that the current distribution was largely restricted to Texas in the USA whereas our predictions show high relative probability of occurrence with higher certainty in both Texas and the neighbouring Mexican states of Coahuila, Nuevo Leon and Tamaulipas, in agreement with the predictions made in 2015 by the Mexican Atlas of Triatomines [24]. The most comprehensive collection of species distribution maps is provided by the Mexican Atlas of Triatomines [24] which generated predictive maps for 19 species of which *T. rubida*, *T.gerstaeckeri*, *T. longipennis*, *T. mexicana*, *T. barberi*, *T. pallidipennis*, *T. mazzottii* and *T. protracta* were also modelled in our study. A visual comparison shows a reasonable alignment between the predictions made by the Mexican Atlas of Triatomines and our results for these eight species within Mexico. It is also interesting to note that our results for *T. dimidiata*, within Mexico, most closely align to the Mexican Atlas of Triatomines’s results for haplogroup 2 of this species [24].

Arboleda *et al*. [28] produced a predictive map of the geographical distribution of *R. pallescens* across Central and South America. Our two studies show broad agreement in Central America but the earlier study predicts high environmental suitability for this species in areas much further south than the region where we predict high probability of occurrence. This result demonstrates a key difference in the two methods used because Arboleda *et al*. quantified associations with environmental variables only whereas we also incorporated a spatial component (the Gaussian process) in our model. Consequently, the earlier study identified locations that were predicted to be suitable much further south than any known reports of this species. Parra-Henao *et al*. [20] also used ecological niche modelling, which they applied to *P. geniculatus*, *R. pallescens*, *R. prolixus* and *T. maculata* in the Caribbean, Pacific, Eastern Plains, Andean and Amazon regions of Colombia. The closest alignment between the results of their study and ours can be seen for *T. maculata*. Both studies predict occurrence of this species in non-contiguous areas of northern Colombia; one from the Guarjira Peninsula heading southwest, and another on the eastern slope of Eastern Cordillera towards the Orinoquía region.

Gurgel-Goncalves *et al*. [26] modelled the ecological niches of 16 triatomine species in Brazil, of which 11 were also modelled in our study. Of these, a visual comparison shows that there is good broad agreement between the 2012 study and our results within Brazil for ten of these species (*P. megistus*, *P. lutzi*, *R. nasutus*, *R. neglectus*, *R. pictipes*, *R. robustus*, *T. brasiliensis*, *T. pseudomaculata*, *T. rubrovaria*, *T. sordida*), however, our results show a lower probability of occurrence for *P. geniculatus* in southeast Brazil whereas the 2012 study predicts presence across this region. Carbajal de la Fuente *et al*. [27] also modelled the potential geographic distribution of *T. pseudomaculata* in 2008 and again our results show good broad agreement.

In conclusion, the maps generated by this study provide a robust summary of the contemporary distributions of the most commonly reported vector species across the Chagas endemic zone. It is important that these maps are viewed within the context of the behaviour and vectorial capacity of each of these species. Summaries of the literature published to-date are provided here and the earlier studies show that most of these triatomine species are potentially important vectors of *T. cruzi* to humans. Each of the indicators of vectorial capacity summarised at a species level here may vary within the range of the species, as well as between species [76, 77]. It is therefore important to map spatial variation in these characteristics, as well as in the species themselves, in order to identify where regions of high vectorial transmission risk are likely to exist.

## Supporting information

S1 File Table of environmental variables. This file contains Table A that describes each covariate that went into the models including the time period for which data was available.
(DOCX)

S2 File Sources of evidence for variables linked to vectorial capacity. Tables B and C provide the full information summarised in Table 3 together with citations for the sources of evidence used.
(DOCX)

S3 File Importance of each covariate to the species models. Table D provides the three most important covariate contributions for each species model. Table E ranks the environmental covariates by their relative contributions across all 30 species models. Figure A shows the relative contribution of each covariate to each species model.
(DOCX)
