# Supplementary material for: Modelling geospatial distributions of the triatomine vectors of Trypanosoma cruzi in Latin America
Source: PLoS Negl Trop Dis. 2020 Aug 10;14(8):e0008411. doi: 10.1371/journal.pntd.0008411 (PMC7440660; doi:10.1371/journal.pntd.0008411)
Supplement: S1 File — This file contains Table A that describes each covariate that went into the models including the time period for which data was available. (DOCX) [file pntd.0008411.s001.docx]

**S1 File: Full description of the environmental variables available to each triatomine species distribution model.**

Table A: Each environmental variable provided to the species models is described in full and the source of the data for each variable is given in the reference list.

| **Title** | **Description** | **Ref.** |
| --- | --- | --- |
| Daytime temperature | Annual daytime land surface temperature from satellite measurements (2000 to 2014). | 1 |
| Nighttime temperature | Annual nighttime land surface temperature from satellite measurements (2000 to 2014). | 1 |
| Diurnal temperature difference | Annual diurnal difference in land surface temperature from satellite measurements (2000 to 2014). | 1 |
| Tasseled cap wetness | Surface wetness from satellite measurements (2000 to 2014). | 2 |
| Tasseled cap brightness | Surface wetness in areas of bare soils and senescent vegetation from satellite measurements (2000 to 2014). | 2 |
| Rainfall | Total annual precipitation using satellite measurements (2000 to 2018). | 3 |
| Elevation | Elevation measured using the hydrologically conditioned Digital Elevation Model. | 4 |
| Slope | Slope of the land measured using the hydrologically conditioned Digital Elevation Model. | 4 |
| Land cover | Proportional cover for each of the International Geosphere and Biosphere Programme (IGBP) land cover classifications:  - water;  - evergreen needleleaf forest (canopy cover >60% and height > 2m);  - evergreen broadleaf forest (canopy cover >60% and height > 2m);  - deciduous needleleaf forest (canopy cover >60% and height > 2m);  - deciduous broadleaf forest (canopy cover >60% and height > 2m);  - mixed forest (canopy cover >60% and height > 2m);  - closed shrubland (woody vegetation <2m tall and shrub canopy cover >60%);  - open shrubland (woody vegetation <2m tall and shrub canopy cover 10-60%);  - woody savanna (trees 30-60% and understory vegetation);  - savanna (trees 10-30% and understory vegetation);  - grassland (herbaceous cover with trees/shrubs <10%);  - permanent wetland (a permanent mixture of water and vegetation over extensive areas);  - cropland (temporary crops with harvest period or bare soil);  - urban and built-up areas;  - cropland-natural vegetation mosaic (mosaic of cropland, forest, - shrubland or grassland);  - snow and ice;  - barren or sparsely populated areas.  Based on satellite measurements (2001-2013). | 5 |
| Vegetation index | Enhanced vegetation index based on satellite measurements (2000 to 2014). | 6 |
| Urbanicity | A binary map of urban areas featuring man-made building structures with a vertical component in 2011. | 7 |
| Nighttime lights | Annual nighttime light intensity based on satellite measurements. | 8 |
| Human population | Population count consistent with national censuses and population registers. | 9 |
| Accessibility | Travel time to the nearest city, measured using human movement and density data, as another proxy for rural, peri-urban and urban areas. | 10 |

**References**

1. Wan Z, Hook S. MOD11A2 MODIS/Terra Land Surface Temperature/Emissivity 8-Day L3 Global 1km SIN Grid V006. 2015. doi:10.5067/modis/mod11a2.006.
2. Lobser SE & Cohen WB (2007) MODIS tasselled cap: land cover characteristics expressed through transformed MODIS data, International Journal of Remote Sensing, 28:22.
3. Funk C, Peterson P, Landsfeld M, Pedreros D, Verdin J, Shukla S, et al. The climate hazards infrared precipitation with stations: a new environmental record for monitoring extremes. Scientific Data. 2015; 2:150066. doi:10.1038/sdata.2015.66.
4. Jarvis A, Reuter HI, Nelson A, Guevara E. Hole-filled SRTM for the globe Version 4. CGIAR-CSI SRTM 90m Database. 2008. http://srtm.csi.cgiar.org. Accessed 23 March 2018.
5. M Friedl DSM. MCD12Q1 MODIS/Terra+Aqua Land Cover Type Yearly L3 Global 500m SIN Grid V006; 2015. Available from: https://lpdaac.usgs.gov/node/1260.
6. Didan K, Munoz AB, Solano R, Huete A (2015) MODIS Vegetation Index User’s Guide (MOD13 Series), University of Arizona, <https://vip.arizona.edu/documents/MODIS/MODIS_VI_UsersGuide_June_2015_C6.pdf>.
7. Esch T, Bachofer F, Heldens W, Hirner A, Marconcini M, Palacios-Lopez D, et al. Where we live: a summary of the achievements and planned evolution of the global urban footprint. Remote Sensing. 2018;10:10.
8. Earth Observation Group. Version 1 VIIRS Day/Night Band Nighttime Lights. https://eogdata.mines.edu/download_dnb_composites.html.
9. Center for International Earth Science Information Network (2018) Gridded Population of the World, Version 4 (GPWv4): Population Count, Revision 11. https://eogdata.mines.edu/download_dnb_composites.html.
10. Weiss DJ, Nelson A, Gibson HS, Temperley W, Peedell S, Lieber A, et al. A global map of travel time to cities to assess inequalities in accessibility in 2015. Nature. 2018;553(7688):333{336. doi:10.1038/nature25181.
