# Supplementary material for: Modelling geospatial distributions of the triatomine vectors of Trypanosoma cruzi in Latin America
Source: PLoS Negl Trop Dis. 2020 Aug 10;14(8):e0008411. doi: 10.1371/journal.pntd.0008411 (PMC7440660; doi:10.1371/journal.pntd.0008411)
Supplement: S2 File — Tables B and C provide the full information summarised in Table 3 together with citations for the sources of evidence used. (DOCX) [file pntd.0008411.s002.docx]

**Table B**. Mean infection prevalence is given with the number of studies that test ≥20 individuals in parentheses. If less than ten studies tested ≥20 individuals, no mean is given but the presence or absence of infected individuals is noted. Defecation intervals were not derived using a standard protocol so no values are given but data sources are listed. Confirmation of human blood meals is noted and evidence for a role in food/drink contamination is listed.

| **Species** | **Mean infection prevalence** | **Defecation interval** | **Human blood meals** | **Implicated in food / drink contamination** |
| --- | --- | --- | --- | --- |
| *Eratyrus mucronatus* | Not found [1, 2] | [3] |  |  |
| *Panstrongylus chinai* | Yes [1] | [4] |  |  |
| *Panstrongylus geniculatus* | 0.076 (46) [1] |  | Yes [5, 6] |  |
| *Panstrongylus lutzi* | 0.068 (27) [1] |  | Yes [7] |  |
| *Panstrongylus megistus* | 0.083 (261) [1] |  | Yes [5, 7] |  |
| *Panstrongylus rufotuberculatus* | Yes [1] |  |  |  |
| *Psammolestes tertius* | Not found [1, 7-9] |  |  |  |
| *Rhodnius nasutus* | 0.085 (73) [1] |  |  |  |
| *Rhodnius neglectus* | 0.040 (120) [1] | [10] | Yes [5] |  |
| *Rhodnius pallescens* | Yes [1] |  | Yes [6, 11] |  |
| *Rhodnius pictipes* | 0.246 (36) [1] |  | Yes [6] | [12] |
| *Rhodnius prolixus* | 0.090 (10) [1] |  | Yes [6, 13] |  |
| *Rhodnius robustus* | 0.259 (19) [1] | [10] |  |  |
| *Triatoma barberi* | Yes [1] | [14] [15] |  |  |
| *Triatoma brasiliensis* | 0.032 (918) [1] | [16] | Yes [7, 17] | [18] |
| *Triatoma dimidiata* | 0.289 (24) [1] |  | Yes [6, 19-22] |  |
| *Triatoma gerstaeckeri* | Yes [1] | [23] | Yes [24] |  |
| *Triatoma guasayana* | Yes [1] |  | Yes [25] |  |
| *Triatoma infestans* | 0.276 (61) [1] | [26] | Yes [27-30] |  |
| *Triatoma longipennis* | 0.556 (14) [1] |  |  |  |
| *Triatoma maculata* | 0.184 (18) [1] |  | Yes [6, 31] |  |
| *Triatoma mazzottii* | Yes [32] [1] | [14] |  |  |
| *Triatoma mexicana* | Yes [1] | [33] |  |  |
| *Triatoma pallidipennis* | 0.480 (17) [1] | [15] | Yes [34] |  |
| *Triatoma protracta* | Yes [1] | [23, 35] |  |  |
| *Triatoma pseudomaculata* | 0.027 (988) [1] |  | Yes [5, 7] |  |
| *Triatoma rubida* | Yes [1] | [35] |  |  |
| *Triatoma rubrovaria* | 0.031 (17) [1] | [36] |  |  |
| *Triatoma sanguisuga* | Yes [1] |  | Yes [37] |  |
| *Triatoma sordida* | 0.008 (1407) [1] |  | Yes [5, 30, 38] |  |

**Table C**. Evidence for colonisation of homes, invasion of homes and invasion of urban areas is listed. Evidence for non-domestic habitats is listed, however, few wild environments have been surveyed.

| **Species** | **Colonises homes** | **Invades homes** | **Urban areas** | **Habitats** |
| --- | --- | --- | --- | --- |
| *Eratyrus mucronatus* | Yes [39] | Yes [40, 41] |  | Native trees [3], Bat colonies, Xenarthra nests, Mud walls [39], Brick piles [42] |
| *Panstrongylus chinai* | Yes [43] | Yes [43, 44] |  | Livestock pens [43, 44], Hen coops [45] |
| *Panstrongylus geniculatus* |  | Yes [2, 5, 6, 40, 46-49] | Yes [50-53] | Palm trees [54], Rodent nests [55], Xenarthra nests [55], Burrows [56] |
| *Panstrongylus lutzi* |  | Yes [7, 57-59] |  | Roof tile piles [60], Hollow trees [61], Burrows [62] |
| *Panstrongylus megistus* | Yes [63] | Yes [5, 7, 47, 57] | Yes [51, 64] | Hen coops [63], Livestock shelter [65] |
| *Panstrongylus rufotuberculatus* | Yes [43] | Yes [43, 66] |  | Domestic [43], Walls [67, 68], Native trees [69] |
| *Psammolestes tertius* |  |  |  | Birds nests [8, 70], Palm trees [71] |
| *Rhodnius nasutus* |  | Yes [57, 72] |  | Palm trees [58, 70, 73], Hen coops [74] |
| *Rhodnius neglectus* |  | Yes [2, 5, 47] | Yes [75] | Palm trees [70, 76], Birds nests [9, 77-79] |
| *Rhodnius pallescens* |  | Yes [6, 31] |  | Palm trees [70, 80-82] |
| *Rhodnius pictipes* |  | Yes [2, 6, 83] | yes [80] | Palm trees [84, 85] |
| *Rhodnius prolixus* | Yes [49, 86, 87] | Yes [6, 40, 49] |  | Palm trees [40, 54, 70, 88-90] |
| *Rhodnius robustus* |  | Yes [2, 48, 49] |  | Palm trees [70, 85] |
| *Triatoma barberi* | Yes [91] | Yes [92, 93] | yes [94] | Poultry coops [95], Brick piles [96], Rodent nests [97] |
| *Triatoma brasiliensis* | Yes [57, 98] | Yes [7, 57-59, 99] |  | Livestock shelters [17, 58, 100], Hen coops [58, 100], Rock piles [17, 58, 100], Cacti [101], Wood piles [74] |
| *Triatoma dimidiata* | Yes [19] | Yes [6, 19] |  | Palm trees [11, 81], Sylvatic [82], Caves [21], Hen coops [102] |
| *Triatoma gerstaeckeri* | Yes [103, 104] | Yes [104-106] |  | Kennels [105, 107] |
| *Triatoma guasayana* |  | Yes [108] |  | Sylvatic [109], Peridomestic [109] |
| *Triatoma infestans* | Yes [29, 110] | Yes [29, 110] |  | Domestic [28, 29], Hen coops [29], Livestock shelters [29], Rocky outcrops [28], Hollow trees [56] |
| *Triatoma longipennis* | Yes [93] | Yes [111, 112] | yes [94] | Rocks, Animal shelters [96, 112], Brick piles [93, 96], Hen coops [95, 96], Wood piles [96] |
| *Triatoma maculata* |  | Yes [6, 31, 40, 49] | Yes [113] | Palm trees [54, 70, 88] |
| *Triatoma mazzottii* |  | Yes [97] |  | Rock piles [97], Rodent nests [97] |
| *Triatoma mexicana* |  | yes [91, 114] |  | Peridomestic [91] |
| *Triatoma pallidipennis* | Yes [115] | Yes [97, 116] | Yes [115] | Bat caves [97] |
| *Triatoma protracta* | Yes [117, 118] | Yes [105, 118] | Yes [119] | Rodent nests [117, 120, 121] |
| *Triatoma pseudomaculata* | Yes [57] | Yes [5, 7, 57, 59, 99] | Yes [52, 53] | Birds nests [58], Hen coops [52, 58], Perches [74], Palm trees [122] |
| *Triatoma rubida* | Yes [118, 121] | Yes [105, 106, 118, 123] | Yes [119] | Peridomestic [105], Rodent nests [121] |
| *Triatoma rubrovaria* |  | Yes [53] | Yes [53] | Rocks [53] |
| *Triatoma sanguisuga* |  | Yes [105, 106, 118, 124] | Yes [125] | Peridomestic [105], Kennels [107], Hen coops [124] |
| *Triatoma sordida* | Yes [126-128] | Yes [5, 47, 52, 129] | Yes [51-53, 127] | Hen coops [128, 129], Birds nests [129], Livestock shelters [128, 129], Coati tree nests [130], Wood [128], Palm trees [71, 122], Hollow trees [56] |

**References**

1. Browne AJ, Guerra CA, Alves RV, da Costa VM, Wilson AL, Pigott DM, et al. The contemporary distribution of Trypanosoma cruzi infection in humans, alternative hosts and vectors (vol 4, 170050, 2017). Scientific Data. 2017;4. doi: 10.1038/sdata.2017.71. PubMed PMID: WOS:000402314300001.

2. Brito RN, Goria DE, Diotaiuti L, Gomes ACF, Souza RCM, Abad-Franch F. Drivers of house invasion by sylvatic Chagas disease vectors in the Amazon-Cerrado transition: A multi-year, state-wide assessment of municipality-aggregated surveillance data. Plos Neglected Tropical Diseases. 2017;11(11). doi: 10.1371/journal.pntd.0006035. PubMed PMID: WOS:000416832800022.

3. Silva Monte GL, Tadei WP, Farias TM. Ecoepidemiology and biology of Eratyrus mucronatus Stal, 1859 (Hemiptera: Reduviidae: Triatominae), a sylvatic vector of Chagas disease in the Brazilian Amazon. Revista Da Sociedade Brasileira De Medicina Tropical. 2014;47(6):723-7. doi: 10.1590/0037-8682-0263-2014. PubMed PMID: WOS:000348204000008.

4. Mosquera KD, Villacis AG, Grijalva MJ. Life Cycle, Feeding, and Defecation Patterns of Panstrongylus chinai (Hemiptera: Reduviidae: Triatominae) Under Laboratory Conditions. Journal of Medical Entomology. 2016;53(4):776-81. doi: 10.1093/jme/tjw027. PubMed PMID: WOS:000379204800005.

5. Minuzzi-Souza TTC, Silva LR, Hagstroem L, Hecht M, Nitz N, Gurgel-Goncalves R. Molecular bloodmeal analyses reveal that Trypanosoma cruzi-infected, native triatomine bugs often feed on humans in houses in central Brazil. Medical and Veterinary Entomology. 2018;32(4):504-8. doi: 10.1111/mve.12324. PubMed PMID: WOS:000449714300014.

6. Hernandez C, Salazar C, Brochero H, Teheran A, Stella Buitrago L, Vera M, et al. Untangling the transmission dynamics of primary and secondary vectors of Trypanosoma cruzi in Colombia: parasite infection, feeding sources and discrete typing units. Parasites & Vectors. 2016;9. doi: 10.1186/s13071-016-1907-5. PubMed PMID: WOS:000388903000004.

7. Araujo Silva MB, de Menezes KR, Guerra de Farias MC, Andrade MS, Alves Victor CC, Lorosa ES, et al. Description of the feeding preferences of triatominae in the Chagas disease surveillance study for the State of Pernambuco, Brazil (Hemiptera: Reduviidae). Revista Da Sociedade Brasileira De Medicina Tropical. 2017;50(4):543-6. doi: 10.1590/0037-8682-0334-2016. PubMed PMID: WOS:000411577300017.

8. Diotaiuti LG, Bezerra CM, Soares CJ, Costa LM, Barbosa SE. DESCRIPTION OF AN AMAZING NEST OF CAATINGA CACHALOTE Pseudoseisura cristata INFESTED BY Psammolestes tertius IN TAUA, STATE OF CEARA, NORTHEASTERN BRAZIL. Revista de Patologia Tropical. 2018;47(2):125-31. doi: 10.5216/rpt.v47i2.54214. PubMed PMID: BCI:BCI201800764506.

9. Gurgel-Goncalves R, Duarte MA, Ramalho ED, Palma ART, Romana CA, Cuba-Cuba CA. Spatial distribution of Triatominae populations (Hemiptera: Reduviidae) in Mauritia flexuosa palm trees in Federal District of Brazil. Revista Da Sociedade Brasileira De Medicina Tropical. 2004;37(3):241-7. doi: 10.1590/s0037-86822004000300010. PubMed PMID: ZOOREC:ZOOR14009050665.

10. Barreto-Santana D, Starling J, Gurgel-Goncalves R, Cuba Cuba CA. Comparative biology and feeding behavior of Rhodnius neglectus and Rhodnius robustus (Triatominae) under laboratory conditions. Revista Da Sociedade Brasileira De Medicina Tropical. 2011;44(4):490-5. doi: 10.1590/s0037-86822011000400018. PubMed PMID: WOS:000294128200018.

11. Saldana A, Pineda V, Martinez I, Santamaria G, Maria Santamaria A, Miranda A, et al. A New Endemic Focus of Chagas Disease in the Northern Region of Veraguas Province, Western Half Panama, Central America. Plos One. 2012;7(4). doi: 10.1371/journal.pone.0034657. PubMed PMID: WOS:000305345200012.

12. Barbosa RL, Dias VL, Lorosa ES, Costa EdG, Pereira KS, Gilioli R, et al. Virulence of Trypanosoma cruzi from vector and reservoir in in natura acai pulp resulting in food-borne acute Chagas disease at Para State, Brazil. Experimental Parasitology. 2019;197:68-75. doi: 10.1016/j.exppara.2018.10.012. PubMed PMID: WOS:000459522500011.

13. Pena-Garcia VH, Gomez-Palacio AM, Triana-Chavez O, Mejia-Jaramillo AM. Eco-Epidemiology of Chagas Disease in an Endemic Area of Colombia: Risk Factor Estimation, Trypanosoma cruzi Characterization and Identification of Blood-Meal Sources in Bugs. American Journal of Tropical Medicine and Hygiene. 2014;91(6):1116-24. doi: 10.4269/ajtmh.14-0112. PubMed PMID: WOS:000345879200008.

14. Martinez-Ibarra JA, Nogueda-Torres B, Gonzalez EP, Alejandre-Aguilar R, Solorio-Cibrian M, Barreto SP, et al. Development of Triatoma rubida sonoriana, Triatoma barberi, and Meccus mazzottii (Heteroptera, Reduviidae) under laboratory conditions. Journal of the American Mosquito Control Association. 2005;21(3):310-5. doi: 10.2987/8756-971x(2005)21[310:dotrst]2.0.co;2. PubMed PMID: WOS:000231955600012.

15. Schettino PMS, Arteaga ID, Bravo MC. Importance of three vectors of Trypanosoma cruzi in Mexico. Medicina-Buenos Aires. 2005;65(1):63-9. PubMed PMID: WOS:000228492100012.

16. Folly-Ramos E, Dornak LL, Orsolon G, Monte Goncalves TC, Lilioso M, Costa J, et al. Vector capacity of members of Triatoma brasiliensis species complex: The need to extend Chagas disease surveillance to Triatoma melanica. Journal of Vector Ecology. 2016;41(1):48-54. doi: 10.1111/jvec.12193. PubMed PMID: WOS:000377219600007.

17. Almeida CE, Faucher L, Lavina M, Costa J, Harry M. Molecular Individual-Based Approach on Triatoma brasiliensis: Inferences on Triatomine Foci, Trypanosoma cruzi Natural Infection Prevalence, Parasite Diversity and Feeding Sources. Plos Neglected Tropical Diseases. 2016;10(2). doi: 10.1371/journal.pntd.0004447. PubMed PMID: WOS:000372567300055.

18. Vargas A, Alves Siqueira Malta JM, da Costa VM, Claudio LDG, Alves RV, Cordeiro GdS, et al. Investigation of an outbreak of acute Chagas disease outside the Amazon Region, in Rio Grande do Norte State, Brazil, 2016. Cadernos De Saude Publica. 2018;34(1). doi: 10.1590/0102-311x00006517. PubMed PMID: WOS:000424383200001.

19. Asuncion Lima-Cordon R, Stevens L, Solorzano Ortiz E, Anaite Rodas G, Castellanos S, Rodas A, et al. Implementation science: Epidemiology and feeding profiles of the Chagas vector Triatoma dimidiata prior to Ecohealth intervention for three locations in Central America. Plos Neglected Tropical Diseases. 2018;12(11). doi: 10.1371/journal.pntd.0006952. PubMed PMID: WOS:000452162500056.

20. Orantes LC, Monroy C, Dorn PL, Stevens L, Rizzo DM, Morrissey L, et al. Uncovering vector, parasite, blood meal and microbiome patterns from mixed-DNA specimens of the Chagas disease vector Triatoma dimidiata. Plos Neglected Tropical Diseases. 2018;12(10). doi: 10.1371/journal.pntd.0006730. PubMed PMID: WOS:000449318100017.

21. Stevens L, Carlota Monroy M, Guadalupe Rodas A, Dorn PL. Hunting, Swimming, and Worshiping: Human Cultural Practices Illuminate the Blood Meal Sources of Cave Dwelling Chagas Vectors (Triatoma dimidiata) in Guatemala and Belize. Plos Neglected Tropical Diseases. 2014;8(9). doi: 10.1371/journal.pntd.0003047. PubMed PMID: WOS:000342796600005.

22. Bustamante DM, De Urioste-Stone SM, Juarez JG, Pennington PM. Ecological, Social and Biological Risk Factors for Continued Trypanosoma cruzi Transmission by Triatoma dimidiata in Guatemala. Plos One. 2014;9(8). doi: 10.1371/journal.pone.0104599. PubMed PMID: WOS:000341127500020.

23. Alejandro Martinez-Ibarra J, Alejandre-Aguilar R, Paredes-Gonzalez E, Alejandro Martinez-Silva M, Solorio-Cibrian M, Nogueda-Torres B, et al. Biology of three species of North American Triatominae (Hemiptera : reduviidae : Triatominae) fed on rabbits. Memorias Do Instituto Oswaldo Cruz. 2007;102(8):925-30. doi: 10.1590/s0074-02762007000800006. PubMed PMID: WOS:000252497600006.

24. Gorchakov R, Trosclair LP, Wozniak EJ, Feria PT, Garcia MN, Gunter SM, et al. Trypanosoma cruzi Infection Prevalence and Bloodmeal Analysis in Triatomine Vectors of Chagas Disease From Rural Peridomestic Locations in Texas, 2013-2014. Journal of Medical Entomology. 2016;53(4):911-8. doi: 10.1093/jme/tjw040. PubMed PMID: WOS:000379204800022.

25. Lucero DE, Ribera W, Carlos Pizarro J, Plaza C, Gordon LW, Pena R, Jr., et al. Sources of Blood Meals of Sylvatic Triatoma guasayana near Zurima, Bolivia, Assayed with qPCR and 12S Cloning. Plos Neglected Tropical Diseases. 2014;8(12). doi: 10.1371/journal.pntd.0003365. PubMed PMID: WOS:000346701000040.

26. Lobbia P, Calcagno J, Mougabure-Cueto G. Excretion/defecation patterns in Triatoma infestans populations that are, respectively, susceptible and resistant to deltamethrin. Medical and Veterinary Entomology. 2018;32(3):311-22. doi: 10.1111/mve.12298. PubMed PMID: WOS:000440655500007.

27. Gurtler RE, del Pilar Fernandez M, Carla Cecere M, Cohen JE. Body size and hosts of Triatoma infestans populations affect the size of bloodmeal contents and female fecundity in rural northwestern Argentina. Plos Neglected Tropical Diseases. 2017;11(12). doi: 10.1371/journal.pntd.0006097. PubMed PMID: WOS:000419108500020.

28. Buitrago R, Bosseno M-F, Depickere S, Waleckx E, Salas R, Aliaga C, et al. Blood meal sources of wild and domestic Triatoma infestans (Hemiptera: Reduviidae) in Bolivia: connectivity between cycles of transmission of Trypanosoma cruzi. Parasites & Vectors. 2016;9. doi: 10.1186/s13071-016-1499-0. PubMed PMID: WOS:000375038500001.

29. Laura Carbajal-de-la-Fuente A, Mariana Provecho Y, del Pilar Fernandez M, Victoria Cardinal M, Lencina P, Spillmann C, et al. The eco-epidemiology of Triatoma infestans in the temperate Monte Desert ecoregion of mid-western Argentina. Memorias Do Instituto Oswaldo Cruz. 2017;112(10):698-708. doi: 10.1590/0074-02760160519. PubMed PMID: WOS:000411575900007.

30. Provecho YM, Sol Gaspe M, del Pilar Fernandez M, Enriquez GF, Weinberg D, Guertler RE. The peri-urban interface and house infestation with Triatoma infestans in the Argentine Chaco: an underreported process? Memorias Do Instituto Oswaldo Cruz. 2014;109(7):923-34. doi: 10.1590/0074-0276140225. PubMed PMID: WOS:000345430900012.

31. Cantillo-Barraza O, Garces E, Gomez-Palacio A, Cortes LA, Pereira A, Marcet PL, et al. Eco-epidemiological study of an endemic Chagas disease region in northern Colombia reveals the importance of Triatoma maculata (Hemiptera: Reduviidae), dogs and Didelphis marsupialis in Trypanosoma cruzi maintenance. Parasites & Vectors. 2015;8. doi: 10.1186/s13071-015-1100-2. PubMed PMID: WOS:000361519200003.

32. Vidal-Acosta V, Ibanez-Bernal S, Martinez-Campos C. Natural infection of Triatominae bugs in Mexican dwellings. Salud Publica De Mexico. 2000;42(6):496-503. doi: 10.1590/s0036-36342000000600005. PubMed PMID: WOS:000166583600008.

33. Martinez-Ibarra JA, Salazar-Schettino PM, Solorio-Cibrian M, Bravo MC, Novelo-Lopez M, Vences MO, et al. Influence of temperature and humidity on the biology of Triatoma mexicana (Hemiptera: Reduviidae: Triatominae) under laboratory conditions. Memorias Do Instituto Oswaldo Cruz. 2008;103(7):719-23. doi: 10.1590/s0074-02762008000700015. PubMed PMID: WOS:000261418000015.

34. Ramsey JM, Gutierrez-Cabrera AE, Salgado-Ramirez L, Peterson AT, Sanchez-Cordero V, Ibarra-Cerdena CN. Ecological Connectivity of Trypanosoma cruzi Reservoirs and Triatoma pallidipennis Hosts in an Anthropogenic Landscape with Endemic Chagas Disease. Plos One. 2012;7(9). doi: 10.1371/journal.pone.0046013. PubMed PMID: WOS:000309517300075.

35. Alejandro Martinez-Ibarra J, Paredes-Gonzalez E, Angel Licon T, Dante Montanez-Valdez O, Rocha-Chavez G, Nogueda-Torres B. The biology of three Mexican-American species of Triatominae (Hemiptera: Reduviidae): Triatoma recurva, Triatoma protracta and Triatoma rubida. Memorias Do Instituto Oswaldo Cruz. 2012;107(5):659-63. doi: 10.1590/s0074-02762012000500013. PubMed PMID: WOS:000306973400013.

36. Almeida CE, Folly-Ramos E, Agapito-Souza R, Magno-Esperanca G, Pacheco RS, Costa J. Triatoma rubrovaria (Blanchard, 1843) (Hemiptera, Reduviidae, Triatominae) IV - bionomic aspects on the vector capacity of nymphs. Memorias Do Instituto Oswaldo Cruz. 2005;100(3):231-5. doi: 10.1590/s0074-02762005000300002. PubMed PMID: WOS:000229743300002.

37. Waleckx E, Suarez J, Richards B, Dorn PL. Triatoma sanguisuga Blood Meals and Potential for Chagas Disease, Louisiana, USA. Emerging Infectious Diseases. 2014;20(12):2141-3. doi: 10.3201/eid2012.131576. PubMed PMID: WOS:000345729900032.

38. Alevi KCC, Rodas LAC, Tartarotti E, Azeredo-Oliveira MTV, Guirado MM. Entoepidemiology of Chagas disease in the Western region of the State of Sao Paulo from 2004 to 2008, and cytogenetic analysis in Rhodnius neglectus (Hemiptera, Triatominae). Genetics and Molecular Research. 2015;14(2):5775-84. doi: 10.4238/2015.May.29.9. PubMed PMID: WOS:000357189800005.

39. Depickere S, Duran P, Lopez R, Martinez E, Chavez T. After five years of chemical control: Colonies of the triatomine Eratyrus mucronatus are still present in Bolivia. Acta Tropica. 2012;123(3):234-8. doi: 10.1016/j.actatropica.2012.05.005. PubMed PMID: WOS:000306769000015.

40. Manuel Angulo-Silva V, Zulay Castellanos-Dominguez Y, Florez-Martinez M, Esteban-Adarme L, Perez-Mancipe W, Elvira Farfan-Garcia A, et al. Human Trypanosomiasis in the Eastern Plains of Colombia: New Transmission Scenario. American Journal of Tropical Medicine and Hygiene. 2016;94(2):348-51. doi: 10.4269/ajtmh.15-0406. PubMed PMID: WOS:000369465500017.

41. Obara MT, Cardoso AdS, Goncalves Pinto MC, de Souza CR, Albuquerque e Silva R, Gurgel-Goncalves R. Eratyrus mucronatus Stal, 1859 (Hemiptera: Reduviidae: Triatominae): First report in the State of Acre, Brazil, and updated geographic distribution in South America. Check List. 2013;9(4):851-4. PubMed PMID: ZOOREC:ZOOR15001001509.

42. Manuel Angulo V, Esteban L, Paola Luna K. Attalea butyracea palms adjacent to housing as a source of infestation by Rhodnius prolixus (Hemiptera: Reduviidae). Biomedica. 2012;32(2):277-85. PubMed PMID: WOS:000208835700016.

43. Grijalva MJ, Villacis AG, Ocana-Mayorga S, Yumiseva CA, Moncayo AL, Baus EG. Comprehensive Survey of Domiciliary Triatomine Species Capable of Transmitting Chagas Disease in Southern Ecuador. Plos Neglected Tropical Diseases. 2015;9(10). doi: 10.1371/journal.pntd.0004142. PubMed PMID: WOS:000364459600047.

44. Solís H, Huamán A, Ferrer A, Tarqui K, Fajardo N, Rojas M, et al. Comunicación preliminar sobre la presencia de Trypanosoma cruzi en departamentos del norte y nororiente del Perú Northeast departments. Anales de la Facultad de Medicina. 2012;73(1):43-6. PubMed PMID: SCIELO:S1025-55832012000100008.

45. Patterson JS, Barbosa SE, Dora Feliciangeli M. On the genus Panstrongylus Berg 1879: Evolution, ecology and epidemiological significance. Acta Tropica. 2009;110(2-3):187-99. doi: 10.1016/j.actatropica.2008.09.008. PubMed PMID: WOS:000266181200009.

46. Ayala Hoyos CJ, HernÁNdez Mendoza CM, Eyes Escalante M, Romero Ricardo LR, ÁLvarez RodrÍGuez RA, Blanco TuirÁN P. DETECCIÓN DE INFECCIÓN NATURAL POR Trypanosoma cruzi (TRYPANOSOMATIDAE) EN TRIATOMINOS DEL MUNICIPIO DE COLOSÓ, COLOMBIA in Triatomines From the Municipality of Colosó, Colombia. Acta Biológica Colombiana. 2019;24(1):180-4. doi: 10.15446/abc.v24n1.72306. PubMed PMID: SCIELO:S0120-548X2019000100180.

47. Ferro e Silva AM, Sobral-Souza T, Vancine MH, Muylaert RL, de Abreu AP, Pelloso SM, et al. Spatial prediction of risk areas for vector transmission of Trypanosoma cruzi in the State of Parana, southern Brazil. Plos Neglected Tropical Diseases. 2018;12(10). doi: 10.1371/journal.pntd.0006907. PubMed PMID: WOS:000449318100074.

48. Rojas-Cortez M, Pinazo M-J, Garcia L, Arteaga M, Uriona L, Gamboa S, et al. Trypanosoma cruzi-infected Panstrongylus geniculatus and Rhodnius robustus adults invade households in the Tropics of Cochabamba region of Bolivia. Parasites & Vectors. 2016;9. doi: 10.1186/s13071-016-1445-1. PubMed PMID: WOS:000372438900007.

49. Garcia-Jordan N, Berrizbeitia M, Luis Concepcion J, Aldana E, Caceres A, Quinones W. Entomological study of Trypanosoma cruzi vectors in the rural communities of Sucre state, Venezuela. Biomedica. 2015;35(2):247-57. doi: 10.7705/biomedica.v35i2.2390. PubMed PMID: WOS:000359178200013.

50. Nakad Bechara CC, Carlos Londono J, Segovia M, Leon Sanchez MA, Martinez P CE, Rodriguez R MM, et al. Genetic variability of Panstrongylus geniculatus (Reduviidae: Triatominae) in the Metropolitan District of Caracas, Venezuela. Infection Genetics and Evolution. 2018;66:236-44. doi: 10.1016/j.meegid.2018.09.011. PubMed PMID: WOS:000452589900031.

51. Ceretti-Junior W, Vendrami DP, de Matos-Junior MO, Rimoldi-Ribeiro A, Alvarez JV, Marques S, et al. Occurrences of triatomines (Hemiptera: Reduviidae) and first reports of Panstrongylus geniculatus in urban environments in the city of Sao Paulo, Brazil. Revista Do Instituto De Medicina Tropical De Sao Paulo. 2018;60. doi: 10.1590/s1678-9946201860033. PubMed PMID: WOS:000439782700003.

52. Mendonca VJ, de Oliveira J, Rimoldi A, Ferreira Filho JCR, de Araujo RF, da Rosa JA. Triatominae Survey (Hemiptera: Reduviidae: Triatominae) in the South-Central Region of the State of Bahia, Brazil between 2008 and 2013. American Journal of Tropical Medicine and Hygiene. 2015;92(5):1076-80. doi: 10.4269/ajtmh.14-0556. PubMed PMID: WOS:000354074900035.

53. Ribeiro AR, Mendonca VJ, Alves RT, Martinez I, de Araujo RF, Mello F, et al. Trypanosoma cruzi strains from triatomine collected in Bahia and Rio Grande do Sul, Brazil. Revista De Saude Publica. 2014;48(2):296-303. doi: 10.1590/s0034-8910.2014048004719. PubMed PMID: WOS:000337102300012.

54. Jacome-Pinilla D, Hincapie-Penaloza E, Ortiz MI, David Ramirez J, Guhl F, Molina J. Risks associated with dispersive nocturnal flights of sylvatic Triatominae to artificial lights in a model house in the northeastern plains of Colombia. Parasites & Vectors. 2015;8. doi: 10.1186/s13071-015-1209-3. PubMed PMID: WOS:000365330600001.

55. Briceño Z, Orlandoni G, Torres E, Mogollón A, Concepción JL, Rodríguez-Bonfante C C, et al. Factores de riesgo asociadas a la enfermedad Chagas en comunidades rurales en Lara, Venezuela Lara State, Venezuela. Revista Costarricense de Salud Pública. 2014;23(1):13-24. PubMed PMID: SCIELO:S1409-14292014000100004.

56. Waleckx E, Depickere S, Salas R, Aliaga C, Monje M, Calle H, et al. New Discoveries of Sylvatic Triatoma infestans (Hemiptera: Reduviidae) Throughout the Bolivian Chaco. American Journal of Tropical Medicine and Hygiene. 2012;86(3):455-8. doi: 10.4269/ajtmh.2012.11-0205. PubMed PMID: WOS:000301166000014.

57. de Barros Vasconcelos Fidalgo ASO, da Costa AC, da Silva Filho JD, Candido DdS, Freitas EC, Pereira LdS, et al. Insect vectors of Chagas disease (Trypanosoma cruzi) in Northeastern Brazil. Revista Da Sociedade Brasileira De Medicina Tropical. 2018;51(2):174-82. doi: 10.1590/0037-8682-0408-2017. PubMed PMID: WOS:000432390600009.

58. Barbosa-Silva AN, Jacome da Camara AC, Martins K, Nunes DF, Camara de Oliveira PI, Medeiros de Azevedo PR, et al. Characteristics of Triatomine infestation and natural Trypanosoma cruzi infection in the State of Rio Grande do Norte, Brazil. Revista Da Sociedade Brasileira De Medicina Tropical. 2016;49(1):57-67. doi: 10.1590/0037-8682-0300-2015. PubMed PMID: WOS:000372848300009.

59. Lima MM, Carvalho-Costa FA, Toma HK, Borges-Pereira J, de Oliveira TG, Sarquis O. Chagas disease and housing improvement in northeastern Brazil: a cross-sectional survey. Parasitology Research. 2015;114(5):1687-92. doi: 10.1007/s00436-015-4350-1. PubMed PMID: WOS:000352723200005.

60. Sarquis O, Sposina R, de Oliveira TG, Mac Cord JR, Cabello PH, Borges-Pereira J, et al. Aspects of peridomiciliary ecotopes in rural areas of Northeastern Brazil associated to triatomine (Hemiptera, Reduviidae) infestation, vectors of Chagas disease. Memorias Do Instituto Oswaldo Cruz. 2006;101(2):143-7. doi: 10.1590/s0074-02762006000200005. PubMed PMID: WOS:000237198000005.

61. Garcia M, Souza L, de Souza RDM, Paula AS, Borges EC, Barbosa SE, et al. Occurrence and variability of Panstrongylus lutzi in the State of Ceara, Brazil. Revista Da Sociedade Brasileira De Medicina Tropical. 2005;38(5):410-5. doi: 10.1590/s0037-86822005000500010. PubMed PMID: WOS:000231921200010.

62. Dias-Lima AG, Menezes D, Sherlock I, Noireau F. Wild habitat and related fauna of Panstrongylus lutzi (Reduviidae, Triatominae). Journal of Medical Entomology. 2003;40(6):989-90. doi: 10.1603/0022-2585-40.6.989. PubMed PMID: WOS:000188185400036.

63. Belisario CJ, Leite Dias JV, Diotaiuti L. Profile of the Trypanosoma cruzi vector infestation in Jaboticatubas, State of Minas Gerais, Brazil. Revista Da Sociedade Brasileira De Medicina Tropical. 2013;46(6):779-82. doi: 10.1590/0037-8682-1712-2013. PubMed PMID: WOS:000330539200020.

64. Lima Ribeiro Castro MA, de Souza Castro GV, de Souza JL, de Souza CR, Ramos LJ, de Oliveira J, et al. First report of Panstrongylus megistus (Hemiptera, Reduviidae, Triatominae) in the State of Acre and Rondonia, Amazon, Brazil. Acta Tropica. 2018;182:158-60. PubMed PMID: ZOOREC:ZOOR15410064088.

65. Machado de Assis GF, de Mello Azeredo BV, Gorla D, Diotaiuti L, de lana M. Entomological surveillance of Chagas disease in Berilo municipality, Jequitinhonha Valley, State of Minas Gerais, Brazil. Revista Da Sociedade Brasileira De Medicina Tropical. 2009;42(6):615-21. doi: 10.1590/s0037-86822009000600001. PubMed PMID: WOS:000273645500001.

66. Avendano-Rangel F, Rey K, Aldana E, Lizano E. First record of Panstrongylus rufotuberculatus (Hemiptera: Reduviidae) in Merida State, Venezuela. Boletin De Malariologia Y Salud Ambiental. 2014;54(2):257-60. PubMed PMID: WOS:000352859500015.

67. Coriano H, Navas C, De Sousa L, Ferrer E, Herrera L. Panstrongylus rufotuberculatus (Hemiptera, Reduviidae, Triatominae) infectado con Trypanosoma cruzi en el estado Anzoátegui (Venezuela) infected with Trypanosoma cruzi in the state of Anzoátegui (Venezuela) Antonio Morocoima. Boletin De Malariologia Y Salud Ambiental. 2012;52(1):135-8. PubMed PMID: SCIELO:S1690-46482012000100014.

68. Morocoima A, Coriano H, Navas C, De Sousa L, Ferrer E, Herrera L. Panstrongylus rufotuberculatus (Hemiptera, Reduviidae, Triatominae) infected with Trypanosoma cruzi in the state of Anzoategui (Venezuela). Boletin de la Direccion de Malariologia y Saneamiento Ambiental. 2012;52(1):135-8. PubMed PMID: ZOOREC:ZOOR15002007393.

69. Salomon OD, Ripoll CM, Rivetti E, Carcavello RU. Presence of Panstrongylus rufotuberculatus (Champion, 1899) (Hemiptera: Reduviidae: Triatominae) in Argentina. Memorias Do Instituto Oswaldo Cruz. 1999;94:285-8. doi: 10.1590/S0074-02761999000300002.

70. Abad-Franch F, Lima MM, Sarquis O, Gurgel-Goncalves R, Sanchez-Martin M, Calzada J, et al. On palms, bugs, and Chagas disease in the Americas. Acta Tropica. 2015;151:126-41. doi: 10.1016/j.actatropica.2015.07.005. PubMed PMID: WOS:000364797900013.

71. Gurgel-Goncalves R, Cura C, Schijman AG, Cuba Cuba CA. Infestation of Mauritia flexuosa palms by triatomines (Hemiptera: Reduviidae), vectors of Trypanosoma cruzi and Trypanosoma rangeli in the Brazilian savanna. Acta Tropica. 2012;121(2):105-11. doi: 10.1016/j.actatropica.2011.10.010. PubMed PMID: WOS:000300132800006.

72. de Souza Coutinho CF, Souza-Santos R, Daflon Teixeira NF, Georg I, Gomes TF, Boia MN, et al. An entomoepidemiological investigation of Chagas disease in the state of Ceara, Northeast Region of Brazil. Cadernos De Saude Publica. 2014;30(4):785-93. doi: 10.1590/0102-311x00176512. PubMed PMID: WOS:000337144300011.

73. Peretolchina T, Pavan MG, Correa-Antonio J, Gurgel-Goncalves R, Lima MM, Monteiro FA. Phylogeography and demographic history of the Chagas disease vector Rhodnius nasutus (Hemiptera: Reduviidae) in the Brazilian Caatinga biome. Plos Neglected Tropical Diseases. 2018;12(9). doi: 10.1371/journal.pntd.0006731. PubMed PMID: WOS:000446054600018.

74. Sarquis O, Carvalho-Costa FA, Toma HK, Georg I, Burgoa MR, Lima MM. Eco-epidemiology of Chagas disease in northeastern Brazil: Triatoma brasiliensis, T. pseudomaculata and Rhodnius nasutus in the sylvatic, peridomestic and domestic environments. Parasitology Research. 2012;110(4):1481-5. doi: 10.1007/s00436-011-2651-6. PubMed PMID: WOS:000302814500020.

75. Cortio Correa Rodrigues VL, Pauliquevis Junior C, da Silva RA, Valerio Wanderley DM, Guirardo MM, Colebrusco Rodas LA, et al. COLONIZATION OF PALM TREES BY Rhodnius neglectus AND HOUSEHOLD AND INVASION IN AN URBAN AREA, ARACATUBA, SAO PAULO STATE, BRAZIL. Revista Do Instituto De Medicina Tropical De Sao Paulo. 2014;56(3):213-8. doi: 10.1590/s0036-46652014000300006. PubMed PMID: WOS:000339315500006.

76. Vianna EN, de Andrade AJ, Stehling Dias FB, Diotaiuti L. The exotic palm Roystonea oleracea (Jacq.) OF Cook as a rural biotype for Rhodnius neglectus Lent, 1954, in Cacu, State of Goias. Revista Da Sociedade Brasileira De Medicina Tropical. 2014;47(5):642-5. doi: 10.1590/0037-8682-0221-2013. PubMed PMID: WOS:000345915700019.

77. Carvalho DB, Almeida CE, Rocha CS, Gardim S, Mendonca VJ, Ribeiro AR, et al. A novel association between Rhodnius neglectus and the Livistona australis palm tree in an urban center foreshadowing the risk of Chagas disease transmission by vectorial invasions in Monte Alto City, Sao Paulo, Brazil. Acta Tropica. 2014;130:35-8. doi: 10.1016/j.actatropica.2013.10.009. PubMed PMID: WOS:000331506900005.

78. Gurgel-Goncalves R, Cuba Cuba CA. Predicting the Potential Geographical Distribution of Rhodnius neglectus (Hemiptera, Reduviidae) Based on Ecological Niche Modeling. Journal of Medical Entomology. 2009;46(4):952-60. doi: 10.1603/033.046.0430. PubMed PMID: WOS:000267623800031.

79. Gurgel-Goncalves R, Cuba CAC. Population structure of Rhodnius neglectus Lent and Psammolestes tertius Lent & Jurberg (Hemiptera, Reduviidae) in bird nests (Fumariidae) on Mauritia flexuosa palm trees in Federal District of Brazil. Revista Brasileira De Zoologia. 2007;24(1):157-63. doi: 10.1590/s0101-81752007000100019. PubMed PMID: WOS:000245370300019.

80. Rodriguez IG, Saldana A, Gonzalez K, Pineda V, Perea M, Santamaria AM, et al. Trypanosoma cruzi Infection in Rhodnius pallescens (Heteroptera: Reduviidae) Infesting Coyol Palms in the Dry Arch of Panama. Journal of Medical Entomology. 2018;55(3):691-700. doi: 10.1093/jme/tjx249. PubMed PMID: WOS:000438231600023.

81. Salcedo-Rivera GA, Herazo-Vitola FY, Cruz JF, Sierra-Serrano O. Fauna associated with the Wine Palm Attalea butyracea (Mutis ex L.f.) Wess.Boer (1988) (Arecales: Arecaceae) in an agroecosystem of Galeras, Sucre, Colombia. Biota Colombiana. 2018;19(1):39-48. PubMed PMID: ZOOREC:ZOOR15412088628.

82. Rodriguez IG, Loaiza JR. American trypanosomiasis, or Chagas disease, in Panama: a chronological synopsis of ecological and epidemiological research. Parasites & Vectors. 2017;10. doi: 10.1186/s13071-017-2380-5. PubMed PMID: WOS:000412693600002.

83. Cabrera R, Vega S, Valderrama Y, Cabanillas K, Fernandez C, Rodriguez O, et al. New focus of active transmission of Chagas disease in indigenous populations in the Peruvian Amazon basin. Revista Da Sociedade Brasileira De Medicina Tropical. 2013;46(3):367-72. doi: 10.1590/0037-8682-1195-2013. PubMed PMID: WOS:000322551300022.

84. Fe NF, Magalhaes LK, Fe FA, Arakian SK, Monteiro WM, Vale Barbosa MdG. Occurrences of triatomines in wild and domestic environments in the municipality of Manaus, State of Amazonas. Revista Da Sociedade Brasileira De Medicina Tropical. 2009;42(6):642-6. doi: 10.1590/s0037-86822009000600006. PubMed PMID: WOS:000273645500006.

85. Ricardo-Silva AH, Lopes CM, Ramos LB, Marques WA, Mello CB, Duarte R, et al. Correlation between populations of Rhodnius and presence of palm trees as risk factors for the emergence of Chagas disease in Amazon region, Brazil. Acta Tropica. 2012;123(3):217-23. doi: 10.1016/j.actatropica.2012.05.008. PubMed PMID: WOS:000306769000012.

86. Montenegro D, Vera M, Zuleta L, Llanos V, Junqueira A. Strategy for determining a baseline in areas of vector interruption for Chagas disease. Revista Panamericana De Salud Publica-Pan American Journal of Public Health. 2016;39(6):341-51. PubMed PMID: WOS:000388448900004.

87. Cordovez JM, Guhl F. The impact of landscape transformation on the reinfestation rates of Rhodnius prolixus in the Orinoco Region, Colombia. Acta Tropica. 2015;151:73-9. doi: 10.1016/j.actatropica.2015.07.030. PubMed PMID: WOS:000364797900007.

88. Morocoima A, Barroeta R, Virguez M, Roschman-Gonzalez A, Chique JD, Ferrer E, et al. Triatominae in Palm Trees and Their Natural Infection by Trypanosoma cruzi in Regions of Eastern Venezuela. Revista peruana de medicina experimental y salud publica. 2018;35(4):563-72. doi: 10.17843/rpmesp.2018.354.3871. PubMed PMID: MEDLINE:30726414.

89. Urbano P, Hincapie E, Manuel Angulo V, Esteban L, Alberto Nunez-Avellaneda L. Population variation of Rhodnius prolixus (Reduviidae: Triatominae) in Attalea butyracea (Arecaceae) in the Colombian Orinoquia region. Revista Colombiana De Entomologia. 2018;44(2):211-6. doi: 10.25100/socolen.v44i2.7315. PubMed PMID: WOS:000454926300011.

90. Urbano P, Poveda C, Molina J. Effect of the physiognomy of Attalea butyracea (Arecoideae) on population density and age distribution of Rhodnius prolixus (Triatominae). Parasites & Vectors. 2015;8. doi: 10.1186/s13071-015-0813-6. PubMed PMID: WOS:000352344000002.

91. Becerril MA, Angeles-Perez V, Noguez-Garcia JC, Imbert-Palafox JL. Transmission Risk of Trypanosoma cruzi in Metztitlan Municipality from Hidalgo State, Mexico, by Characterization of Domiciliary Units and their Entomologic Indexes. Neotropical Entomology. 2010;39(5):810-7. doi: 10.1590/s1519-566x2010000500021. PubMed PMID: WOS:000284502300021.

92. Rivas N, Gonzalez-Guzman S, Alejandre-Aguilar R. First record of Triatoma barberi Usinger, 1939 (Hemiptera: Reduviidae) in northern State of Mexico, Mexico. Journal of Vector Ecology. 2018;43(2):337-9. doi: 10.1111/jvec.12319. PubMed PMID: WOS:000449478600016.

93. Breniere SF, Pietrokosky S, Gastelum EM, Bosseno MF, Soto MM, Ouaissi A, et al. Feeding patterns of Triatoma longipennis Usinger (Hemiptera, Reduviidae) in peridomestic habitats of a rural community in Jalisco State, Mexico. Journal of Medical Entomology. 2004;41(6):1015-20. doi: 10.1603/0022-2585-41.6.1015. PubMed PMID: WOS:000225274700003.

94. Gomez-Hernandez C, Rezende-Oliveira K, Cortes Zarate A, Cortes Zarate E, Trujillo-Contreras F, Ramirez LE. Prevalence of triatomines (Hemiptera : Reduviidae : Triatominae) infected by Trypanosoma cruzi: seasonality and distribution in the Cienega region of the State of Jalisco, Mexico. Revista Da Sociedade Brasileira De Medicina Tropical. 2008;41(3):257-62. doi: 10.1590/s0037-86822008000300007. PubMed PMID: WOS:000258695700007.

95. Alejandro Martinez-Ibarra J, Alejandro Martinez-Grant J, Roberto Verdugo-Cervantes M, Bustos-Saldana R, Nogueda-Torres B. Monitoring triatomid bug (Hemiptera: Reduviidae) presence by sentinel chicken coops in Southern Jalisco State, Mexico. Biomedica. 2010;30(1):140-5. doi: 10.7705/biomedica.v30i1.161. PubMed PMID: WOS:000277976500016.

96. Walter A, Lozano-Kasten F, Bosseno M-F, Castillo Ruvalcaba EG, Gutierrez MS, Montano Luna CE, et al. Peridomicilary habitat and risk factors for Triatoma infestation in a rural community of the Mexican occident. American Journal of Tropical Medicine and Hygiene. 2007;76(3):508-15. doi: 10.4269/ajtmh.2007.76.508. PubMed PMID: WOS:000244918700019.

97. Ramsey JM, Ordonez R, Cruz-Celis A, Alvear AL, Chavez V, Lopez R, et al. Distribution of domestic triatominae and stratification of Chagas Disease transmission in Oaxaca, Mexico. Medical and Veterinary Entomology. 2000;14(1):19-30. doi: 10.1046/j.1365-2915.2000.00214.x. PubMed PMID: WOS:000086260100004.

98. dos Santos SM, de Sousa DM, dos Santos JP, Pinheiro do Nascimento Vieira JF, Monte Goncalves TC, dos Santos-Mallet JR, et al. Entomological survey in the state of Piaui, Northeastern Brazil, reveals intradomiciliary colonization of Triatoma brasiliensis macromelasoma. Revista Do Instituto De Medicina Tropical De Sao Paulo. 2017;59. doi: 10.1590/s1678-9946201759027. PubMed PMID: WOS:000403274300003.

99. Bezerra CM, Barbosa SE, Moreira de Souza RdC, Barezani CP, Gurtler RE, Ramos AN, Jr., et al. Triatoma brasiliensis Neiva, 1911: food sources and diversity of Trypanosoma cruzi in wild and artificial environments of the semiarid region of Ceara, northeastern Brazil. Parasites & Vectors. 2018;11. doi: 10.1186/s13071-018-3235-4. PubMed PMID: WOS:000453631900002.

100. Daflon-Teixeira NF, Coutinho C, Gomes TF, Toma HK, Duarte R, Boia MN, et al. Multiple Approaches to Address Potential Risk Factors of Chagas Disease Transmission in Northeastern Brazil. American Journal of Tropical Medicine and Hygiene. 2019;100(2):296-302. doi: 10.4269/ajtmh.18-0480. PubMed PMID: WOS:000460410600015.

101. Valenca-Barbosa C, Lima MM, Sarquis O, Bezerra CM, Abad-Franch F. Short Report: A Common Caatinga Cactus, Pilosocereus gounellei, is an Important Ecotope of Wild Triatoma brasiliensis Populations in the Jaguaribe Valley of Northeastern Brazil. American Journal of Tropical Medicine and Hygiene. 2014;90(6):1059-62. doi: 10.4269/ajtmh.13-0204. PubMed PMID: WOS:000336820200014.

102. Reyes-Novelo E, Ruiz-Pina H, Escobedo-Ortegon J, Barrera-Perez M, Manrique-Saide P, Rodriguez-Vivas RI. Triatoma dimidiata (Latreille) Abundance and Infection with Trypanosoma cruzi in a Rural Community of Yucatan, Mexico. Neotropical Entomology. 2013;42(3):317-24. doi: 10.1007/s13744-013-0120-x. PubMed PMID: WOS:000318801600014.

103. Wozniak EJ, Lawrence G, Gorchakov R, Alamgir H, Dotson E, Sissel B, et al. THE BIOLOGY OF THE TRIATOMINE BUGS NATIVE TO SOUTH CENTRAL TEXAS AND ASSESSMENT OF THE RISK THEY POSE FOR AUTOCHTHONOUS CHAGAS DISEASE EXPOSURE. Journal of Parasitology. 2015;101(5):520-8. doi: 10.1645/15-748. PubMed PMID: WOS:000364147000003.

104. Molina-Garza ZJ, Mercado-Hernandez R, Molina-Garza DP, Galaviz-Silva L. Trypanosoma cruzi-infected Triatoma gerstaeckeri (Hemiptera: Reduviidae) from Nuevo Leon, Mexico, and pathogenicity of the regional strain. Biomedica. 2015;35(3):372-8. doi: 10.7705/biomedica.v35i3.2589. PubMed PMID: WOS:000364260500011.

105. Curtis-Robles R, Hamer SA, Lane S, Levy MZ, Hamer GL. Bionomics and Spatial Distribution of Triatomine Vectors of Trypanosoma cruzi in Texas and Other Southern States, USA. American Journal of Tropical Medicine and Hygiene. 2018;98(1):113-21. doi: 10.4269/ajtmh.17-0526. PubMed PMID: WOS:000430950900022.

106. Curtis-Robles R, Wozniak EJ, Auckland LD, Hamer GL, Hamer SA. Combining Public Health Education and Disease Ecology Research: Using Citizen Science to Assess Chagas Disease Entomological Risk in Texas. Plos Neglected Tropical Diseases. 2015;9(12). doi: 10.1371/journal.pntd.0004235. PubMed PMID: WOS:000368345100022.

107. Curtis-Robles R, Snowden KF, Dominguez B, Dinges L, Rodgers S, Mays G, et al. Epidemiology and Molecular Typing of Trypanosoma cruzi in Naturally-Infected Hound Dogs and Associated Triatomine Vectors in Texas, USA. Plos Neglected Tropical Diseases. 2017;11(1). doi: 10.1371/journal.pntd.0005298. PubMed PMID: WOS:000394152000065.

108. Cavallo MJ, Amelotti I, Gorla DE. Invasion of rural houses by wild Triatominae in the arid Chaco. Journal of Vector Ecology. 2016;41(1):97-102. doi: 10.1111/jvec.12199. PubMed PMID: WOS:000377219600013.

109. Abrahan L, Gorla D, Catala S. Active dispersal of Triatoma infestans and other triatomines in the Argentinean arid Chaco before and after vector control interventions. Journal of Vector Ecology. 2016;41(1):90-6. doi: 10.1111/jvec.12198. PubMed PMID: WOS:000377219600012.

110. Jose Cavallo M, Amelotti I, Abrahan L, Cueto G, Gorla DE. Rural houses infestation by Triatoma infestans in northwestern Argentina: Vector control in a high spatial heterogeneous infestation area. Plos One. 2018;13(8). doi: 10.1371/journal.pone.0201391. PubMed PMID: WOS:000440778600039.

111. Gurgel-Goncalves R, Galvao C, Costa J, Peterson AT. Geographic distribution of chagas disease vectors in Brazil based on ecological niche modeling. Journal of Tropical Medicine. 2012;2012:705326-. doi: 10.1155/2012/705326. PubMed PMID: MEDLINE:22523500.

112. Alejandro Martinez-Ibarra J, Nogueda-Torres B, Dante Montanez-Valdez O, Rocha-Chavez G, Maria Tapia-Gonzalez J. Presence of Meccus longipennis and Triatoma recurva in the state of Durango, Mexico. Boletin De Malariologia Y Salud Ambiental. 2012;52(1):129-33. PubMed PMID: WOS:000328160500013.

113. Ricardo-Silva A, Monte Goncalves TC, Luitgards-Moura JF, Lopes CM, da Silva SP, Bastos AQ, et al. Triatoma maculata colonises urban domicilies in Boa Vista, Roraima, Brazil. Memorias Do Instituto Oswaldo Cruz. 2016;111(11):703-6. doi: 10.1590/0074-02760160026. PubMed PMID: WOS:000388621100007.

114. Becerril-Flores MA, Rangel-Flores E, Imbert-Palafox JL, Gomez-Gomez JV, Figueroa-Gutierrez AH. Human infection and risk of transmission of Chagas disease in Hidalgo State, Mexico. American Journal of Tropical Medicine and Hygiene. 2007;76(2):318-23. doi: 10.4269/ajtmh.2007.76.318. PubMed PMID: WOS:000244142200018.

115. Ramsey JM, Alvear AL, Ordonez R, Munox G, Garcia A, Lopez R, et al. Risk factors associated with house infestation by the Chagas disease vector Triatoma pallidipennis in Cuernavaca metropolitan area, Mexico. Medical and Veterinary Entomology. 2005;19(2):219-28. doi: 10.1111/j.0269-283X.2005.00563.x. PubMed PMID: WOS:000229613200009.

116. Villagran ME, Marin C, Hurtado A, Sanchez-Moreno M, de Diego JA. Natural infection and distribution of triatomines (Hemiptera : Reduviidae) in the state of Queretaro, Mexico. Transactions of the Royal Society of Tropical Medicine and Hygiene. 2008;102(8):833-8. doi: 10.1016/j.trstmh.2008.05.005. PubMed PMID: WOS:000258201600016.

117. Dolhun EP, Antes AW. Case Report: A Case of Cardboard Boxes Likely Facilitating the Biting of a Patient by Trypanosoma cruzi-Infected Triatomine Bugs. American Journal of Tropical Medicine and Hygiene. 2016;95(5):1115-7. doi: 10.4269/ajtmh.16-0455. PubMed PMID: WOS:000400206600029.

118. Klotz SA, Shirazi FM, Boesen K, Beatty NL, Dorn PL, Smith S, et al. Kissing Bug (Triatoma spp.) Intrusion into Homes: Troublesome Bites and Domiciliation. Environmental Health Insights. 2016;10:45-9. doi: 10.4137/ehi.s32834. PubMed PMID: WOS:000387839300001.

119. Reisenman CE, Savary W, Cowles J, Gregory TL, Hildebrand JG. The Distribution and Abundance of Triatomine Insects, Potential Vectors of Chagas Disease, in a Metropolitan Area in Southern Arizona, United States. Journal of Medical Entomology. 2012;49(6):1254-61. doi: 10.1603/me12139. PubMed PMID: WOS:000311303200009.

120. Shender L, Niemela M, Conrad P, Goldstein T, Mazet J. Habitat Management to Reduce Human Exposure to Trypanosoma cruzi and Western Conenose Bugs (Triatoma protracta). Ecohealth. 2016;13(3):525-34. doi: 10.1007/s10393-016-1153-5. PubMed PMID: WOS:000386363400011.

121. Paredes-Gonzalez E, Villa Velarde R, Sotelo Estrada MI, Ortega-Garcia J. DOMESTIC, PERIDOMESTIC AND WILD TRIATOMINES DE TECTION (Hemiptera: reduviidae) IN GUAYMAS, SONORA, MEXICO. Biotecnia. 2015;17(2):3-8. doi: 10.18633/bt.v17i2.172. PubMed PMID: WOS:000383366200001.

122. Gurgel-Goncalves R, Ribeiro Junior G, da Costa EMn. Infestation of Palm Trees by Triatomines (Hemiptera: Reduviidae) in the State of Bahia, Brazil. EntomoBrasilis. 2012;5(3):227-31. PubMed PMID: ZOOREC:ZOOR14908031874.

123. Beatty NL, Perez-Velez CM, Yaglom HD, Carson S, Liu E, Khalpey ZI, et al. Evidence of Likely Autochthonous Transmission of Chagas Disease in Arizona. American Journal of Tropical Medicine and Hygiene. 2018;99(6):1534-6. doi: 10.4269/ajtmh.18-0485. PubMed PMID: WOS:000452400500030.

124. Moudy RM, Michaels S, Jameson SB, Londono B, Lopez V, Caillouet KA, et al. Factors Associated With Peridomestic Triatoma sanguisuga (Hemiptera: Reduviidae) Presence in Southeastern Louisiana. Journal of Medical Entomology. 2014;51(5):1043-50. doi: 10.1603/me13234. PubMed PMID: WOS:000341644600020.

125. Dye-Braumuller KC, Gorchakov R, Gunter SM, Nielsen DH, Roachell WD, Wheless A, et al. Identification of Triatomines and Their Habitats in a Highly Developed Urban Environment. Vector-Borne and Zoonotic Diseases. 2019;19(4):265-73. doi: 10.1089/vbz.2018.2352. PubMed PMID: WOS:000462935600006.

126. Vianna EN, de Paula Souza e Guimaraes RJ, Souza CR, Gorla D, Diotaiuti L. Chagas disease ecoepidemiology and environmental changes in northern Minas Gerais state, Brazil. Memorias Do Instituto Oswaldo Cruz. 2017;112(11):760-8. doi: 10.1590/0074-02760170061. PubMed PMID: WOS:000414023000005.

127. de Camargo FA, Romano CA, Elias CN, Garcia da Silva HH, da Silva IG. SINANTHROPIZATION OF TRIATOMINES (HEMIPTERA: REDUVIIDAE) IN THE ITUMBIARA MUNICIPALITY, GOIAS, BRAZIL. Revista de Patologia Tropical. 2017;46(4):339-42. doi: 10.5216/rpt.v46i4.51015. PubMed PMID: BCI:BCI201800271058.

128. Nogared Rossi JC, Duarte EC, Gurgel-Goncalves R. Factors associated with the occurrence of Triatoma sordida (Hemiptera: Reduviidae) in rural localities of Central-West Brazil. Memorias Do Instituto Oswaldo Cruz. 2015;110(2):192-200. doi: 10.1590/0074-02760140395. PubMed PMID: WOS:000354066400005.

129. Rodriguez-Planes LI, Gaspe MS, Enriquez GF, Gurtler RE. Habitat-Specific Occupancy and a Metapopulation Model of Triatoma sordida (Hemiptera: Reduviidae), a Secondary Vector of Chagas Disease, in Northeastern Argentina. Journal of Medical Entomology. 2018;55(2):370-81. doi: 10.1093/jme/tjx227. PubMed PMID: WOS:000429320000014.

130. de Lima JS, Rocha FL, Alves FM, Lorosa ES, Jansen AM, Mourao GdM. Infestation of arboreal nests of coatis by triatomine species, vectors of Trypanosoma cruzi, in a large Neotropical wetland. Journal of Vector Ecology. 2015;40(2):379-85. doi: 10.1111/jvec.12177. PubMed PMID: WOS:000365721100022.
