# Supplementary material for: Modelling geospatial distributions of the triatomine vectors of Trypanosoma cruzi in Latin America
Source: PLoS Negl Trop Dis. 2020 Aug 10;14(8):e0008411. doi: 10.1371/journal.pntd.0008411 (PMC7440660; doi:10.1371/journal.pntd.0008411)

**S3 File: Importance of each variables to the species models.**

Table D: The three most important variables selected by each species model. Full descriptions of each variable are given in the S1 File. Variables associated with the urban-rural gradient, forest cover and other vegetation cover are highlighted.

| **Vector species** | **Variable 1** | **Variable 2** | **Variable 3** |
| --- | --- | --- | --- |
| *Eratyrus mucronatus* | evergreen broadleaf forest cover | savannah cover | diurnal temperature difference |
| *Panstrongylus chinai* | Gaussian process | vegetation index | accessibility |
| *Panstrongylus geniculatus* | grassland cover | elevation | vegetation index |
| *Panstrongylus lutzi* | daytime temperature | nighttime temperature | diurnal temperature difference |
| *Panstrongylus megistus* | daytime temperature | rainfall | nighttime temperature |
| *Panstrongylus rufotuberculatus* | evergreen broadleaf forest cover | daytime temperature | urbanicity |
| *Psammolestes tertius* | daytime temperature | nighttime temperature | diurnal temperature difference |
| *Rhodnius nasutus* | daytime temperature | nighttime temperature | diurnal temperature difference |
| *Rhodnius neglectus* | Gaussian process | urbanicity | rainfall |
| *Rhodnius pallescens* | daytime temperature | nighttime temperature | diurnal temperature difference |
| *Rhodnius pictipes* | daytime temperature | rainfall | evergreen broadleaf forest cover |
| *Rhodnius prolixus* | Gaussian process | human population count | cropland / vegetation mosaic |
| *Rhodnius robustus* | Gaussian process | nighttime temperature | vegetation index |
| *Triatoma barberi* | Gaussian process | elevation | rainfall |
| *Triatoma brasiliensis* | daytime temperature | nighttime temperature | diurnal temperature difference |
| *Triatoma dimidiata* | Gaussian process | diurnal temperature difference | rainfall |
| *Triatoma gerstaeckeri* | daytime temperature | diurnal temperature difference | nighttime temperature |
| *Triatoma guasayana* | daytime temperature | diurnal temperature difference | nighttime temperature |
| *Triatoma infestans* | daytime temperature | vegetation index | rainfall |
| *Triatoma longipennis* | daytime temperature | diurnal temperature difference | nighttime temperature |
| *Triatoma maculata* | vegetation index | elevation | savannah cover |
| *Triatoma mazzottii* | Gaussian process | urbanicity | rainfall |
| *Triatoma mexicana* | Gaussian process | rainfall | cropland cover |
| *Triatoma pallidipennis* | daytime temperature | nighttime temperature | diurnal temperature difference |
| *Triatoma protracta* | Gaussian process | nighttime temperature | vegetation index |
| *Triatoma pseudomaculata* | Gaussian process | human population count | rainfall |
| *Triatoma rubida* | Gaussian process | elevation | wetness on bare soil |
| *Triatoma rubrovaria* | rainfall | nighttime temperature | elevation |
| *Triatoma sanguisuga* | daytime temperature | diurnal temperature difference | accessibility |
| *Triatoma sordida* | daytime temperature | nighttime temperature | surface wetness |

Table E: Ranked importance of each variable across all 30 species models.

| **Variable** | **Mean contribution** |
| --- | --- |
| daytime temperature | 23.06464766 |
| Gaussian process | 18.68584253 |
| evergreen broadleaf forest cover | 14.16072354 |
| nighttime temperature | 12.80213498 |
| diurnal temperature difference | 8.417579156 |
| vegetation index | 6.998388801 |
| rainfall | 6.771563892 |
| elevation | 4.091039647 |
| accessibility | 1.829492743 |
| surface wetness | 1.640571338 |
| urbanicity | 1.571928209 |
| nighttime lights | 1.568905011 |
| human population count | 1.294679299 |
| cropland cover | 1.252210755 |
| savannah cover | 1.245032532 |
| open shrubland cover | 1.181525143 |
| grassland cover | 1.095434709 |
| cropland / vegetation mosaic | 0.846644409 |
| wetness on bare soil | 0.647827618 |
| woody savannah cover | 0.523741237 |
| slope | 0.268852137 |
| wetland cover | 1.53E-04 |
| deciduous broadleaf forest cover | 1.56E-05 |
| mixed forest cover | 4.36E-06 |
| water | not selected |
| evergreen needleleaf forest cover | not selected |
| deciduous needleleaf forest cover | not selected |
| closed shrubland cover | not selected |
| urban and built-up area cover | not selected |
| snow and ice cover | not selected |
| barren area cover | not selected |
| unclassified land cover | not selected |
| no data on land cover | not selected |

Figure A: Each cell is the relative contribution of a term in the model on the x-axis (term contribution / total contribution of all terms * 100%). The figure must be read row-wise as the total contribution of all terms was calculated on a per species basis. Thus, row-wise contributions of the terms sum up to 100%. We use “term” instead of “variable” or “covariate” here, as one term effect could depend on multiple variables, e.g., for some models, the contribution of the variable elevation is defined by the term f(longitude,latitude)*elevation.


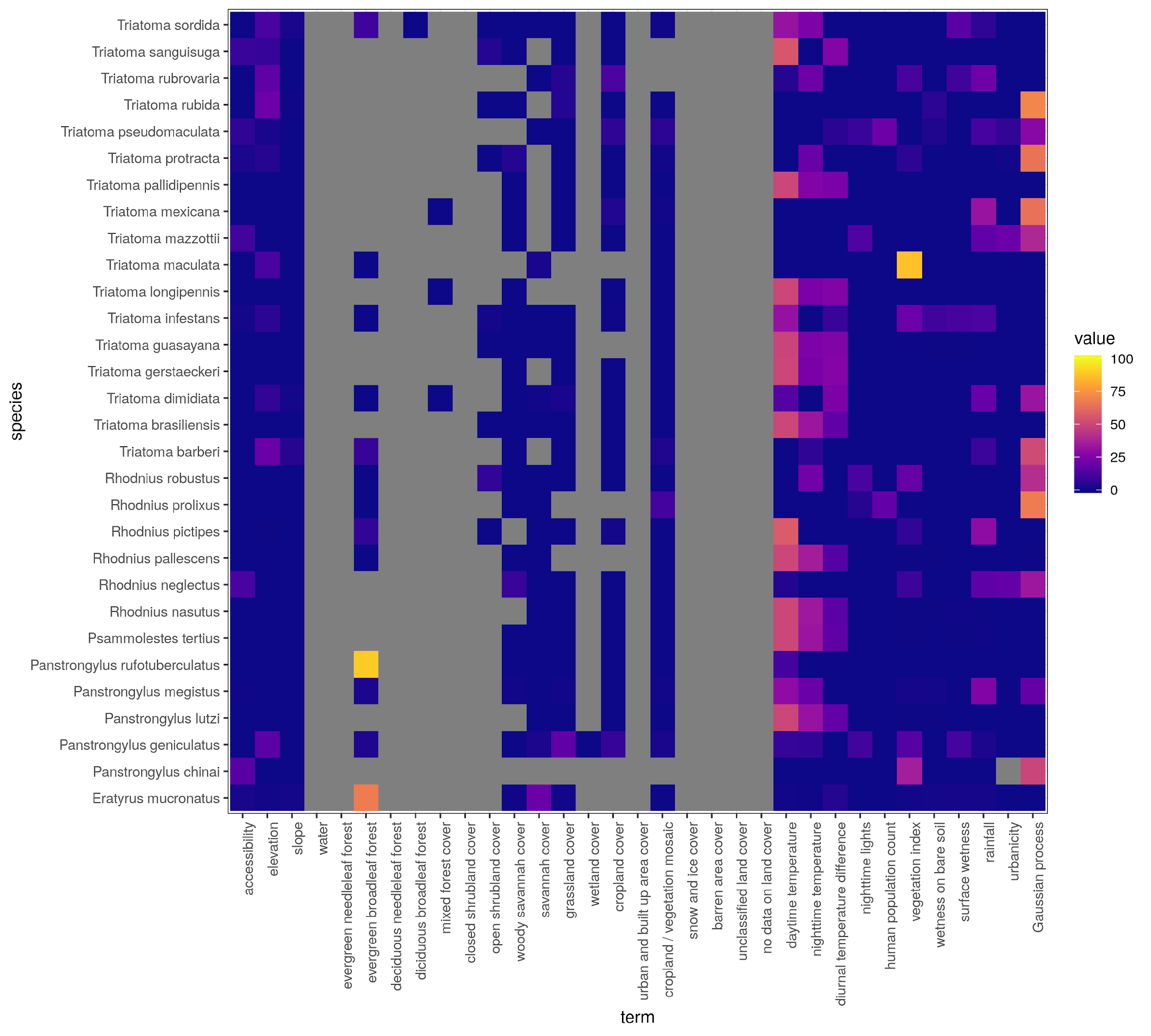

Supplement: S3 File — Table D provides the three most important covariate contributions for each species model. Table E ranks the environmental covariates by their relative contributions across all 30 species models. Figure A shows the relative contribution of each covariate to each species model. (DOCX) [file pntd.0008411.s003.docx]
